# Use of quantitative molecular diagnostic methods to assess the aetiology, burden, and clinical characteristics of diarrhoea in children in low-resource settings: a reanalysis of the MAL-ED cohort study

**DOI:** 10.1016/S2214-109X(18)30349-8

**Published:** 2018-10-01

**Authors:** James A Platts-Mills, Jie Liu, Elizabeth T Rogawski, Furqan Kabir, Paphavee Lertsethtakarn, Mery Siguas, Shaila S Khan, Ira Praharaj, Arinao Murei, Rosemary Nshama, Buliga Mujaga, Alexandre Havt, Irene A Maciel, Timothy L McMurry, Darwin J Operario, Mami Taniuchi, Jean Gratz, Suzanne E Stroup, James H Roberts, Adil Kalam, Fatima Aziz, Shahida Qureshi, M Ohedul Islam, Pimmada Sakpaisal, Sasikorn Silapong, Pablo P Yori, Revathi Rajendiran, Blossom Benny, Monica McGrath, Benjamin J J McCormick, Jessica C Seidman, Dennis Lang, Michael Gottlieb, Richard L Guerrant, Aldo A M Lima, Jose Paulo Leite, Amidou Samie, Pascal O Bessong, Nicola Page, Ladaporn Bodhidatta, Carl Mason, Sanjaya Shrestha, Ireen Kiwelu, Estomih R Mduma, Najeeha T Iqbal, Zulfiqar A Bhutta, Tahmeed Ahmed, Rashidul Haque, Gagandeep Kang, Margaret N Kosek, Eric R Houpt, The MAL-ED Network Investigators, Angel Mendez Acosta, Rosa Rios de Burga, Cesar Banda Chavez, Julian Torres Flores, Maribel Paredes Olotegui, Silvia Rengifo Pinedo, Dixner Rengifo Trigoso, Angel Orbe Vasquez, Imran Ahmed, Didar Alam, Asad Ali, Muneera Rasheed, Sajid Soofi, Ali Turab, Aisha Yousafzai, Anita KM Zaidi, Binob Shrestha, Bishnu Bahadur Rayamajhi, Tor Strand, Geetha Ammu, Sudhir Babji, Anuradha Bose, Ajila T George, Dinesh Hariraju, M. Steffi Jennifer, Sushil John, Shiny Kaki, Priyadarshani Karunakaran, Beena Koshy, Robin P Lazarus, Jayaprakash Muliyil, Preethi Ragasudha, Mohan Venkata Raghava, Sophy Raju, Anup Ramachandran, Rakhi Ramadas, Karthikeyan Ramanujam, Anuradha Rose, Reeba Roshan, Srujan L Sharma, Shanmuga Sundaram, Rahul J Thomas, William K Pan, Ramya Ambikapathi, J Daniel Carreon, Viyada Doan, Christel Hoest, Stacey Knobler, Mark A Miller, Stephanie Psaki, Zeba Rasmussen, Stephanie A Richard, Karen H Tountas, Erling Svensen, Caroline Amour, Eliwaza Bayyo, Regisiana Mvungi, John Pascal, Ladislaus Yarrot, Leah Barrett, Rebecca Dillingham, William A Petri, Rebecca Scharf, AM Shamsir Ahmed, Md Ashraful Alam, Umma Haque, Md Iqbal Hossain, Munirul Islam, Mustafa Mahfuz, Dinesh Mondal, Baitun Nahar, Fahmida Tofail, Ram Krishna Chandyo, Prakash Sunder Shrestha, Rita Shrestha, Manjeswori Ulak, Aubrey Bauck, Robert Black, Laura Caulfield, William Checkley, Gwenyth Lee, Kerry Schulze, Samuel Scott, Laura E Murray-Kolb, A Catharine Ross, Barbara Schaefer, Suzanne Simons, Laura Pendergast, Cláudia B Abreu, Hilda Costa, Alessandra Di Moura, José Quirino Filho, Álvaro M Leite, Noélia L Lima, Ila F Lima, Bruna LL Maciel, Pedro HQS Medeiros, Milena Moraes, Francisco S Mota, Reinaldo B Oriá, Josiane Quetz, Alberto M Soares, Rosa MS Mota, Crystal L Patil, Cloupas Mahopo, Angelina Maphula, Emanuel Nyathi

**Affiliations:** a Division of Infectious Diseases and International Health, University of Virginia, Charlottesville, VA, USA; b Department of Public Health Sciences, University of Virginia, Charlottesville, VA, USA; c Aga Khan University, Karachi, Pakistan; d Armed Forces Research Institute of Medical Sciences (AFRIMS), Bangkok, Thailand; e Asociación Benéfica PRISMA, Iquitos, Peru; f International Centre for Diarrhoeal Disease Research, Dhaka, Bangladesh; g Christian Medical College, Vellore, India; h University of Venda, Thohoyandou, South Africa; i Haydom Global Health Institute, Haydom, Tanzania; j Kilimanjaro Clinical Research Institute, Moshi, Tanzania; k Federal University of Ceara, Fortaleza, Brazil; l Fundação Oswaldo Cruz (Fiocruz), Rio de Janeiro, Brazil; m Bloomberg School of Public Health, Johns Hopkins University, Baltimore, MD, USA; n Fogarty International Center, National Institutes of Health, Bethesda, MD, USA; o Foundation for the National Institutes of Health, Bethesda, MD, USA; p National Institute for Communicable Diseases, Johannesburg, South Africa; q Walter Reed/AFRIMS Research Unit, Nepal, Kathmandu, Nepal; r University of Bergen, Bergen, Norway

## Abstract

**Background:**

Optimum management of childhood diarrhoea in low-resource settings has been hampered by insufficient data on aetiology, burden, and associated clinical characteristics. We used quantitative diagnostic methods to reassess and refine estimates of diarrhoea aetiology from the Etiology, Risk Factors, and Interactions of Enteric Infections and Malnutrition and the Consequences for Child Health and Development (MAL-ED) cohort study.

**Methods:**

We re-analysed stool specimens from the multisite MAL-ED cohort study of children aged 0–2 years done at eight locations (Dhaka, Bangladesh; Vellore, India; Bhaktapur, Nepal; Naushero Feroze, Pakistan; Venda, South Africa; Haydom, Tanzania; Fortaleza, Brazil; and Loreto, Peru), which included active surveillance for diarrhoea and routine non-diarrhoeal stool collection. We used quantitative PCR to test for 29 enteropathogens, calculated population-level pathogen-specific attributable burdens, derived stringent quantitative cutoffs to identify aetiology for individual episodes, and created aetiology prediction scores using clinical characteristics.

**Findings:**

We analysed 6625 diarrhoeal and 30 968 non-diarrhoeal surveillance stools from 1715 children. Overall, 64·9% of diarrhoea episodes (95% CI 62·6–71·2) could be attributed to an aetiology by quantitative PCR compared with 32·8% (30·8–38·7) using the original study microbiology. Viral diarrhoea (36·4% of overall incidence, 95% CI 33·6–39·5) was more common than bacterial (25·0%, 23·4–28·4) and parasitic diarrhoea (3·5%, 3·0–5·2). Ten pathogens accounted for 95·7% of attributable diarrhoea: *Shigella* (26·1 attributable episodes per 100 child-years, 95% CI 23·8–29·9), sapovirus (22·8, 18·9–27·5), rotavirus (20·7, 18·8–23·0), adenovirus 40/41 (19·0, 16·8–23·0), enterotoxigenic *Escherichia coli* (18·8, 16·5–23·8), norovirus (15·4, 13·5–20·1), astrovirus (15·0, 12·0–19·5), *Campylobacter jejuni or C coli* (12·1, 8·5–17·2), *Cryptosporidium* (5·8, 4·3–8·3), and typical enteropathogenic *E coli* (5·4, 2·8–9·3). 86·2% of the attributable incidence for *Shigella* was non-dysenteric. A prediction score for shigellosis was more accurate (sensitivity 50·4% [95% CI 46·7–54·1], specificity 84·0% [83·0–84·9]) than current guidelines, which recommend treatment only of bloody diarrhoea to cover *Shigella* (sensitivity 14·5% [95% CI 12·1–17·3], specificity 96·5% [96·0–97·0]).

**Interpretation:**

Quantitative molecular diagnostics improved estimates of pathogen-specific burdens of childhood diarrhoea in the community setting. Viral causes predominated, including a substantial burden of sapovirus; however, *Shigella* had the highest overall burden with a high incidence in the second year of life. These data could improve the management of diarrhoea in these low-resource settings.

**Funding:**

Bill & Melinda Gates Foundation.

## Introduction

Although diarrhoea mortality has declined substantially since 1990, diarrhoeal incidence and morbidity remain a substantial problem.^1^, ^2^, ^3^ Studies of diarrhoea aetiology have focused on children presenting to care, but this only represents a minority of diarrhoea episodes.^4^ Improved estimates of the aetiology and burden of diarrhoea at the community level could help ameliorate this morbidity by informing treatment guidelines and public health interventions.

The application of molecular diagnostics for enteropathogens has provided increased sensitivity and, through quantification, resolution to identify causes of diarrhoea.^5^, ^6^ These studies have revealed that some pathogens, including *Shigella*, enterotoxigenic *Escherichia coli*, and adenovirus 40/41 have been underestimated as causes of diarrhoea. Outside of cholera-endemic settings, WHO guidelines^7^ recommend antimicrobial therapy only for children with bloody diarrhoea as a surrogate for shigellosis diagnosis. However, in practice antibiotics are used much more broadly,^8^ and the presence of blood in stools is a poor marker of *Shigella* infection.^6^, ^9^, ^10^ Moreover, shigellosis has been associated with mortality independent of dysentery.^10^ Although broader antibiotic treatment of diarrhoea might thus be beneficial, this benefit must be weighed against increasing antibiotic resistance, which is a growing concern and might disproportionately affect children in these settings.^11^, ^12^

**Research in context**

**Evidence before this study**

We searched PubMed for articles published in any language since Jan 1, 1990, using the search terms “(diarrhea OR diarrhoea) AND (etiology OR aetiology OR cause*) AND (pediatric OR paediatric OR infant* OR children) AND cohort AND (PCR OR molecular)”. We identified 370 publications, of which four estimated pathogen-specific diarrhoea burdens for more than a single pathogen from prospective child cohorts. Of these, two used a pan-molecular diagnostic approach: the first study was done in the USA and the second was done at a single site and did not incorporate pathogen quantity.

**Added value of this study**

This study provides a comprehensive, multisite assessment of the aetiology of childhood diarrhoea of any severity using quantitative PCR for a broad range of enteropathogens in the context of home-based surveillance. This approach provided a complete overview of the aetiology and burden of infectious diarrhoea and assessed the value of clinical characteristics to better target syndromic treatment of diarrhoea in these settings. Our study identified several pathogens, including *Shigella*, sapovirus, and adenovirus 40/41, as major aetiologies of childhood diarrhoea that were either missed or the burden of which was substantially underestimated by previous microbiological approaches. These results suggest that incorporation of clinical characteristics other than blood (such as child age) into antibiotic treatment guidelines might be of value.

**Implications of all available evidence**

Ten enteropathogens are responsible for most cases of infectious diarrhoea in children in low-resource settings, including several that were previously underestimated. Because of the high burden of *Shigella*, and because only a minority of shigellosis is dysenteric, guidelines for antibiotic treatment of childhood diarrhoea that focus on dysentery should be reviewed.

We have previously reported diarrhoea aetiology estimates in the Etiology, Risk Factors, and Interactions of Enteric Infections and Malnutrition and the Consequences for Child Health and Development (MAL-ED) multisite birth cohort study,^13^ using qualitative diagnostics to assess a subset of specimens.^14^ In this study, we retested stool specimens from the MAL-ED study using quantitative PCR to refine these aetiology estimates, test for a broader range of pathogens, and identify clinical characteristics that might help discriminate between different causes, guide treatment algorithms, and improve antibiotic use for children with diarrhoea in low-resource settings.

## Methods

### Study design and participants

The MAL-ED study design has been previously described.^13^ Between November, 2009, and February 2012, children were enrolled from the community within 17 days of birth at eight locations: Dhaka, Bangladesh; Vellore, India; Bhaktapur, Nepal; Naushero Feroze, Pakistan; Venda, South Africa; Haydom, Tanzania; Fortaleza, Brazil; and Loreto, Peru. Children were included if their mother was aged 16 years or older, their family intended to remain in the study area for at least 6 months from enrolment, they were from a singleton pregnancy, and they had no other siblings enrolled in the study. Children with a birthweight or enrolment weight of less than 1500 g and children diagnosed with congenital disease or severe neonatal disease were excluded. All sites received ethics approval from their respective governmental, local institutional, and collaborating institutional ethics review boards. Written informed consent was obtained from the parent or guardian of every child.

### Surveillance and sample collection

Fieldworkers visited children twice weekly for surveillance of child illnesses, antibiotic use, and vaccine administration. Diarrhoea was defined as maternal report of three or more loose stools in 24 h, or one stool with visible blood. Episodes separated by 48 h without study-defined diarrhoea were considered distinct episodes. The duration, number of loose stools in 24 h, presence of dehydration, fever, and vomiting were also recorded for each episode of diarrhoea. Dehydration was defined as irritability that was difficult to console, increased thirst, loss of skin turgor, sunken eyes, or lethargy.^7^ Diarrhoea for 7 days or more was considered a prolonged episode, and a high frequency was defined as more than 6 stools in any 24 h period during an episode. A diarrhoea severity score was calculated for every episode as previously described.^14^ Diarrhoeal and monthly non-diarrhoeal surveillance stool specimens were collected. Diarrhoea was considered to be treated by antibiotics if antibiotic use was reported on any day of the diarrhoea episode, and inappropriate antibiotic use was defined post hoc on the basis of a microbiological gold standard as either unnecessary treatment (ie, for non-*Shigella* diarrhoea) or inappropriate antibiotic selection (ie, use of an antibiotic class other than fluoroquinolones or macrolides for *Shigella* diarrhoea).^15^, ^16^, ^17^, ^18^

### Stool testing

All diarrhoeal stools and non-diarrhoeal stools collected for surveillance for months 1–12, 15, 18, 21, and 24 were analysed according to a standardised protocol, as previously described.^14^, ^19^ We used all available diarrhoeal and monthly non-diarrhoeal stool specimens from children who had complete follow-up to age 24 months. We used custom-designed TaqMan Array Cards (Thermo Fisher, Carlsbad, CA, USA) that compartmentalised probe-based quantitative PCR assays for 29 enteropathogens. Assays for *Plesiomonas shigelloides* were included on a subset of cards. All procedures, including assay validation, nucleic acid extraction, quantitative PCR setup, and quality control have been described previously (appendix).^20^, ^21^ Raw stool aliquots were stored at −80°C before extraction. Bacteriophage MS2 was used as an external control to monitor efficiency of nucleic acid extraction and amplification. We included one extraction blank per batch and one no-template amplification control per ten cards to exclude laboratory contamination. The detection of rotavirus was considered false positive if obtained within 28 days of rotavirus vaccine administration. Both *Shigella* and enteroinvasive *E coli* can be detected using the *ipaH* target; however, on the basis of previous findings^6^, ^22^ and for simplicity, we considered the detection of *ipaH* to be consistent with *Shigella* infection.

### Statistical analysis

For all analyses, we used the quantification cycle value as an inverse measure of pathogen quantity, whereby one quantification cycle unit corresponds to a doubling in quantity. A quantification cycle of 35 was considered the analytical limit of detection. We estimated pathogen-specific burdens of diarrhoea by calculating attributable fractions, which incorporate both the frequency of pathogen detection in diarrhoea and the association between pathogen quantity and diarrhoea.^23^ This allowed for differential attribution of aetiology based on the amount of pathogen nucleic acid present. To estimate this association, a generalised linear mixed-effects model (GLMM) was fit for each pathogen, whereby the outcome was diarrhoea, and predictors were the quantity of the modelled pathogen, the quantity of each other pathogen, child sex, test batch, child age in 3 month intervals, an interaction between pathogen quantity and child age, a random slope for each site, and a random intercept for each individual. A quadratic term for the quantity of the modelled pathogen was considered if the prevalence in diarrhoea at any quantity was at least 5% and included if it improved model fit on the basis of the Akaike information criterion. Non-diarrhoeal stools were required to be collected at least 7 days before and after any diarrhoea episode. All pathogens with at least one detection at any quantity in diarrhoeal stools and any association with diarrhoea in single-pathogen analysis (ie, the same model but without adjustment for other pathogens) were included in the final analysis. Attributable fractions were calculated by summing the pathogen attributable fraction for each episode (AFe) across each of *j* episodes with the following equation:

$$\sum_{i=1}^{j}\left(\frac{1}{j}\right)\times AFe_{i}$$

where

AFei=1-(1ORei)

and ORe is the pathogen-specific and quantity-specific odds ratio from the GLMM for each episode. Attributable incidence rates were calculated as the product of the number of episodes identified by surveillance and the attributable fractions divided by the number of child years at risk and expressed as rates per 100 child-years. 95% CIs were estimated by bootstrapping with 1000 iterations.

To estimate the association between pathogen-attributable diarrhoea and clinical characteristics, a single GLMM was fit for each characteristic and included the AFe for each pathogen, child age, a quadratic term for age, and nested random effects for site and individual. Coefficients were scaled to the AFe range for each pathogen. An AFe for non-infectious diarrhoea was defined as one minus the sum of all pathogen-specific AFes with a lower bound of zero. To assess model-based prediction of pathogen-attributable episodes, we first identified aetiologic detections for each episode, using a stringent quantitative cutoff (AFe ≥0·5; appendix). If more than one aetiology was identified, the pathogen with the highest AFe was considered the primary aetiology, and all others were considered secondary aetiologies. We then derived a prediction score from a GLMM, with an outcome of an aetiologic episode, and predictors of blood in stool, fever, prolonged duration, dehydration, vomiting, high stool frequency, and child age in 3 month intervals and nested random effects for site and individual. The fixed effects coefficients were scaled, rounded, and summed. We fit a receiver operating characteristic curve, and the lowest score that achieved at least 80% specificity was selected as the cutoff. For each prediction score, a diarrhoea episode was considered positive if it had a score greater than or equal to the cutoff. The Youden Index^24^ was calculated as sensitivity + specificity – 1, and 95% CIs were calculated using the binomial distribution. All analyses were done in R version 3.4.3.

### Role of the funding source

The funder of the study had no role in study design, data collection, data analysis, data interpretation, or writing of the report. The corresponding author had full access to all the data in the study and had final responsibility for the decision to submit for publication.

## Results

Of 2145 enrolled children, 1715 had complete follow-up to 24 months, from whom 44 570 stools were collected. Of the 44 570 stools collected, 42 630 (95·6%) had sufficient specimen available and 40 406 (90·7%) had valid quantitative PCR results for all pathogens included in the aetiology analysis (table 1; appendix). After excluding non-diarrhoeal stools collected within 7 days of a diarrhoeal episode, and pathogens with no association with diarrhoea in univariate analysis, 6625 diarrhoeal and 30 968 non-diarrhoeal stools and 27 pathogens were examined for aetiology (appendix). The modelled associations between pathogen quantity and diarrhoea were robust for age and diarrhoea severity (appendix). 420 children without complete follow-up were excluded from quantitative PCR testing; using the original microbiological work-up, the prevalence of pathogens in diarrhoea was similar between children who were and were not included in the quantitative PCR testing (appendix).

**Table 1:** Diarrhoea surveillance, sample collection, and stool testing by quantitative PCR in the MAL-ED cohort

	**Children enrolled**	**Children with follow-up to 24 months**	**Diarrhoea episodes reported**	**Diarrhoea stools collected from unique episodes**	**Diarrhoea stools available for testing**	Diarrhoea stools with valid results*	**Surveillance stools collected**	**Surveillance stools available for testing**	Surveillance stools with valid results*	**Surveillance stools included in the aetiology analysis**†
Dhaka, Bangladesh	265	210	1520	1438 (94·6%)	1392 (96·8%)	1374 (98·7%)	4528	4353 (96·1%)	4267 (98·0%)	3787
Vellore, India	251	227	960	722 (75·2%)	675 (93·5%)	623 (92·3%)	5058	4924 (97·4%)	4689 (95·2%)	2767
Bhaktapur, Nepal	240	227	1060	955 (90·1%)	911 (95·4%)	899 (98·7%)	5160	5065 (98·2%)	5011 (98·9%)	4457
Naushero Feroze, Pakistan	277	246	3110	2123 (68·3%)	1871 (88·1%)	1789 (95·6%)	4871	4676 (96·0%)	4499 (96·2%)	4518
Venda, South Africa	314	237	295	179 (60·7%)	147 (82·1%)	113 (76·9%)	5399	5160 (95·6%)	4428 (85·8%)	3458
Haydom, Tanzania	262	209	537	178 (33·1%)	164 (92·1%)	155 (94·5%)	4657	4345 (93·3%)	4033 (92·8%)	3833
Fortaleza, Brazil	233	165	168	117 (69·6%)	100 (85·5%)	88 (88·0%)	3242	2994 (92·4%)	2795 (93·4%)	4291
Loreto, Peru	303	194	1742	1642 (94·3%)	1617 (98·5%)	1584 (98·0%)	4301	4236 (98·5%)	4059 (95·8%)	3857
Total	2145	1715	9392	7354 (78·3%)	6877 (93·5%)	6625 (96·3%)	37 216	35 753 (96·1%)	33 781 (94·5%)	30 968

Data are n or n (%).

* Valid results required for all 27 enteropathogens included in the aetiology analysis (*Plesiomonas* was included on a subset of cards [5015 of 6877 tested diarrhoeal stools and 32 276 of 35 753 tested surveillance stools]).

† For the aetiology analysis, only surveillance stools that were collected at least 7 days both before and after any reported episode of diarrhoea were included.

**Figure 1:**
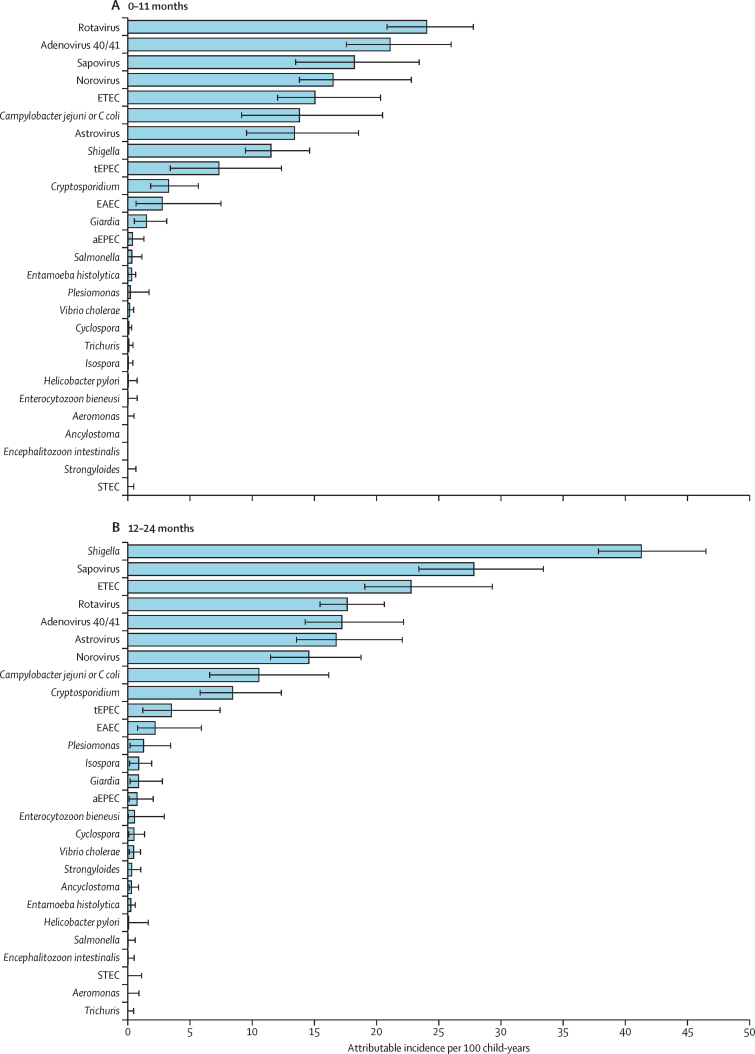
Attributable incidence of pathogen-specific diarrhoea at ages 0–11 months (A) and 12–24 months (B) in the MAL-ED cohort study by quantitative PCR Error bars show 95% CI. ETEC=enterotoxigenic *E coli*. tEPEC=typical enteropathogenic *E coli*. EAEC=enteroaggregative *E coli*. aEPEC=atypical enteropathogenic *E coli.* STEC=Shiga toxin-producing *E coli*.

**Figure 2:**
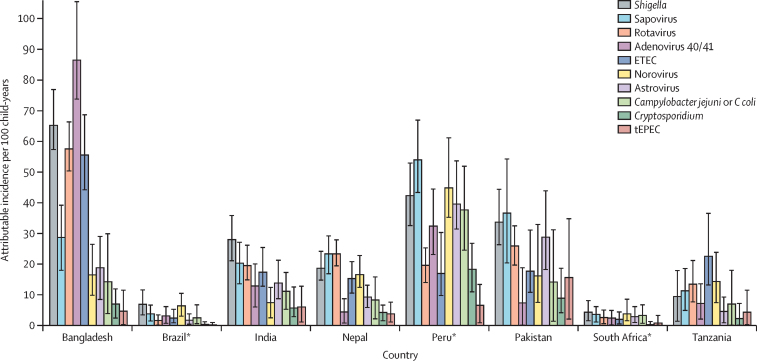
Attributable incidence of pathogen-specific diarrhoea by site in the MAL-ED cohort study by quantitative PCR The ten pathogens with the highest overall attributable incidence are shown. Error bars show 95% CI. ETEC=enterotoxigenic *E coli*. tEPEC=typical enteropathogenic *E coli*. *Sites where rotavirus vaccine has been added to the national immunisation schedule.

In the first year of life, the incidence of diarrhoea was 304·9 episodes per 100 child-years, and rotavirus (23·9 episodes per 100 child-years, 95% CI 20·7–27·6), adenovirus 40/41 (20·9, 17·4–25·8), sapovirus (18·1, 13·4–23·3), norovirus (16·4, 13·7–22·6) and enterotoxigenic *E coli* (14·9, 12·0–20·2) had the highest attributable incidences. In the second year of life, diarrhoea incidence was 242·7 episodes per 100 child-years, and *Shigella* (41·3, 95% CI 37·8–46·5), sapovirus (27·8, 23·4–33·4), enterotoxigenic *E coli* (22·8, 19·1–29·3), rotavirus (17·7, 15·5–20·6) and adenovirus 40/41 (17·2, 14·3–22·2) had the highest attributable incidence (figure 1). Across both years, diarrhoea incidence was 273·8 episodes per 100 child-years, and 95·7% of attributable diarrhoea was attributed to ten pathogens: *Shigella* (26·1 attributable episodes per 100 child-years, 95% CI 23·8–29·9), sapovirus (22·8, 18·9–27·5), rotavirus (20·7, 18·8–23·0), adenovirus 40/41 (19·0, 16·8–23·0), enterotoxigenic *E coli* (18·8, 16·5–23·8), norovirus (15·4, 13·5–20·1), astrovirus (15·0, 12·0–19·5), *Campylobacter jejuni or C coli* (12·1, 8·5–17·2), *Cryptosporidium* (5·8, 4·3–8·3), and typical enteropathogenic *E coli* (5·4, 2·8–9·3). Although there were differences in the pathogen hierarchy by site (figure 2 and appendix), the top five aetiologies at each site came from these ten pathogens with the exception of enteroaggregative *E coli*, which had the third highest incidence in Fortaleza, Brazil (4·0 episodes per 100 child-years, 95% CI 0·2–9·8) and fifth highest in Haydom, Tanzania (9·7, 0·7–28·9). A more stringent definition of non-diarrhoeal stools which excluded those collected within 28 days of diarrhoea increased attributable incidence by more than one episode per 100 child-years for norovirus (2·4 episodes per 100 child-years), *C jejuni or C coli* (2·4), sapovirus (2·0), *Cryptosporidium* (1·9), and enterotoxigenic *E coli* (1·2; appendix). At the time of the study, rotavirus vaccine had been introduced to the national programmes of Brazil, Peru, and South Africa. Rotavirus was responsible for 8·9% of all attributable incidence among countries in which rotavirus vaccine had not been introduced (the second highest pathogen-specific burden) and only 4·2% among countries where rotavirus vaccine had been introduced (the eighth highest pathogen-specific burden). *Shigella* had the highest attributable burden of moderate-to-severe diarrhoea using the definition created for the Global Enteric Multicenter Study,^6^ whereas rotavirus had the highest burden of severe diarrhoea using a modified Vesikari score (appendix).^14^

Overall, 64·9% (95% CI 62·6–71·2) of diarrhoea episodes could be attributed to an infectious aetiology by quantitative PCR versus 32·8% (30·8–38·7) with the original microbiology (appendix). Among the top ten aetiologies, pathogen-attributable diarrhoeal burden was markedly increased for adenovirus 40/41 and *Shigella*, and to a lesser degree for astrovirus, enterotoxigenic *E coli*, and typical EPEC. Sapovirus was not tested for in the original MAL-ED study.^13^ The proportion of diarrhoea that was attributable to pathogens was higher in the second year of life than the first year of life (82·8% [95% CI 78·7–91·1] *vs* 50·5%, [47·2–56·2]). Overall, viral diarrhoea (36·4%, 95% CI 33·6–39·5) was more common than bacterial (25·0%, 23·4–28·4) and parasitic diarrhoea (3·5%, 3·0–5·2). In the second year of life, the proportion of attribution to bacterial diarrhoea (43·3% in the second year *vs* 31·9% in the first year) and parasitic diarrhoea (6·8% *vs* 3·9%) increased, whereas the proportion of attribution to viral diarrhoea decreased (49·9% *vs* 64·2%).

Next, we assessed the association between pathogen attribution and distinguishing clinical characteristics (table 2). *Shigella* diarrhoea was associated with blood in stool, fever, prolonged duration, dehydration, and high frequency, whereas rotavirus diarrhoea was associated with fever, vomiting, dehydration, high frequency, and high severity. All viral causes, with the exception of astrovirus, were associated with vomiting. Non-infectious diarrhoea was associated with the absence of each clinical characteristic with the exception of prolonged duration. Notably, 86·2% of the attributable incidence for *Shigella* was non-dysenteric (appendix).

**Table 2:** Clinical features associated with aetiology-specific diarrhoea

		**Blood in stool (n=315)**	**Fever (n=2170)**	Prolonged duration*(n=1381)	**Dehydration (n=692)**	**Vomiting (n=1778)**	**High frequency**†**(n=1697)**	**Severe**‡§**(n=1120)**
Bacteria								
	*Campylobacter jejuni* or *C coli*	4·53 (2·71–7·57)	1·30 (0·98–1·72)	1·19 (0·82–1·73)	0·59 (0·33–1·09)	0·68 (0·48–0·97)	1·08 (0·76–1·54)	0·69 (0·42–1·11)
	tEPEC	0·24 (0·07–0·87)	1·19 (0·84–1·70)	1·09 (0·69–1·70)	0·94 (0·48–1·84)	0·99 (0·64–1·52)	0·82 (0·53–1·27)	1·36 (0·84–2·19)
	ETEC	0·54 (0·29–1·02)	0·98 (0·77–1·25)	0·96 (0·69–1·34)	1·23 (0·78–1·93)	1·11 (0·87–1·41)	1·21 (0·94–1·57)	1·37 (0·98–1·92)
	Shigella	7·39 (5·20–10·49)	1·32 (1·10–1·58)	1·66 (1·31–2·10)	1·55 (1·11–2·16)	0·81 (0·65–1·02)	1·73 (1·40–2·14)	1·28 (0·96–1·70)
Viruses								
	Adenovirus 40/41	1·06 (0·55–2·06)	1·22 (0·92–1·62)	0·87 (0·58–1·29)	2·14 (1·24–3·69)	1·29 (0·99–1·68)	0·95 (0·70–1·29)	1·36 (0·91–2·01)
	Astrovirus	0·24 (0·09–0·62)	0·92 (0·71–1·20)	0·78 (0·55–1·11)	1·38 (0·90–2·12)	1·10 (0·83–1·46)	1·23 (0·92–1·65)	1·09 (0·75–1·58)
	Norovirus	0·45 (0·20–0·99)	0·79 (0·58–1·07)	0·66 (0·44–1·00)	1·59 (0·95–2·67)	1·81 (1·36–2·42)	0·86 (0·61–1·23)	1·38 (0·91–2·09)
	Rotavirus	0·46 (0·24–0·89)	1·47 (1·24–1·74)	0·79 (0·60–1·04)	3·23 (2·44–4·28)	2·31 (1·97–2·72)	1·66 (1·38–1·99)	2·46 (1·99–3·06)
	Sapovirus	0·39 (0·21–0·74)	0·84 (0·68–1·05)	0·83 (0·62–1·11)	1·21 (0·84–1·74)	1·51 (1·22–1·88)	1·01 (0·79–1·29)	1·12 (0·83–1·53)
Protozoa								
	*Cryptosporidium*	0·25 (0·06–1·00)	1·26 (0·87–1·84)	1·50 (0·93–2·43)	1·57 (0·83–2·95)	1·27 (0·82–1·96)	1·06 (0·64–1·75)	1·29 (0·71–2·33)
No aetiology identified		0·69 (0·50–0·96)	0·84 (0·75–0·96)	1·03 (0·88–1·21)	0·48 (0·38–0·60)	0·61 (0·53–0·70)	0·72 (0·62–0·83)	0·59 (0·49–0·70)

Data are prevalence ratio (95% CI). tEPEC=typical enteropathogenic *E coli.* ETEC=enterotoxigenic *E coli.*

* Diarrhoea for 7 days or longer.

† More than six loose stools in 24 h.

‡ Severity score of 6 or higher.

§ Score derived from components of the Vesikari score.^14^ Analysis includes all episodes of diarrhoea with complete valid quantitative PCR results and clinical characteristics for the ten pathogens listed (n=6676).

**Figure 3:**
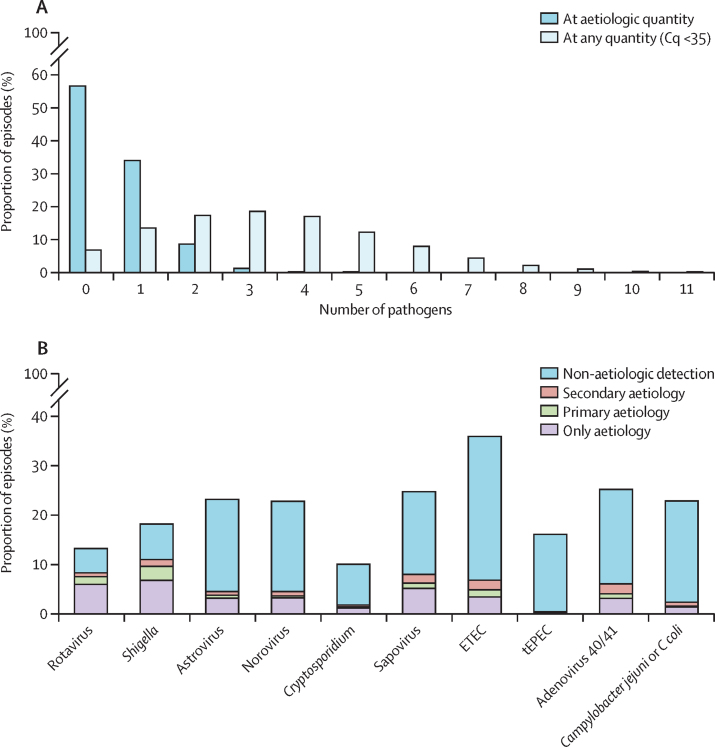
Aetiologic detections and co-infections in diarrhoea episodes (A) Number of pathogens detected in diarrhoea episodes. Aetiologic quantity was defined as an AFe of 0·5 or more. (B) Distribution of pathogen detections, stratified by aetiologic attribution. When more than one aetiologic detection was present, the primary aetiology was defined as the pathogen with the highest AFe. Pathogens are ordered according to the proportion of aetiologic detections for which they were the primary aetiology. Cq=quantification cycles. ETEC=enterotoxigenic *E coli*. tEPEC=typical enteropathogenic *E coli*. AFe=attributable fraction for that episode.

Using detection at any quantity, diarrhoeal stools had a mean of 3·4 pathogens (SD 2·0) compared with 2·5 pathogens (1·8) in non-diarrhoeal stools (p<0·001). Using a stringent quantitative cutoff to identify episode-level aetiology (appendix), 2243 (33·9%) of 6625 episodes had one pathogen, and 644 (9·7%) of 6625 episodes had two or more pathogens (figure 3A). Frequently, no infectious aetiology was identified for diarrhoeal episodes that occurred in the first 6 months of life (appendix). Among episodes with multiple aetiologies, rotavirus and *Shigella* were usually the primary aetiologies, whereas typical enteropathogenic *E coli*, adenovirus 40/41, and *C jejuni or C coli* were often secondary aetiologies (figure 3B). The clinical phenotype of diarrhoea was altered by co-pathogens. Among 1915 episodes with a viral aetiology, co-attribution to *Shigella* was associated with presence of blood (prevalence ratio 12·74, 95% CI 5·94–27·31) and prolonged duration (2·33, 1·44–3·78). Among 1264 episodes with a bacterial aetiology, co-attribution to viral pathogens was associated with the absence of blood (prevalence ratio 0·57, 95% CI 0·34–0·96) and vomiting (1·55, 1·11–2·16).

Because of the high *Shigella* burden, the suspected benefit of treating *Shigella* independent of the presence of blood, and because only a small minority of *Shigella* diarrhoea episodes were dysenteric, we evaluated whether other clinical characteristics could help inform treatment decisions (table 3). We found that a prediction score could identify most cases of *Shigella* diarrhoea (sensitivity 50·4% [95% CI 46·7–54·1], specificity 84·0% [83·0–84·9], area under the curve [AUC] 0·783; table 3) and the prediction scores were consistent across sites with a high burden of disease (appendix). Age was an important determinant; diarrhoeal stools in children aged 6 months or younger were never positive, diarrhoeal stools in children aged between 7–12 months were positive only in the case of bloody diarrhoea, and in the second year of life a broader combination of clinical characteristics was sufficient. Complete score components are shown in the appendix. This *Shigella* score performed better than the presence of blood alone, which is used in current guidelines as an indication for treatment of shigellosis (sensitivity 14·5% [95% CI 12·1–17·3], specificity 96·5% [96·0–97·0], AUC 0·555; table 3). Among *Shigella* diarrhoea episodes, longer duration was associated with maternal report of blood (odds ratio 1·10 per additional day of diarrhoea, 95% CI 1·02–1·18), suggesting bloody diarrhoea was a late manifestation of disease. The performance of the *Shigella* score was slightly improved in the subset of moderate-to-severe diarrhoea (AUC 0·824; appendix). Clinical prediction scores performed less well for other pathogens (table 3; appendix).

**Table 3:** Model-based prediction of diarrhoea aetiology

	**Disease**	**Positive episodes, % (SD)**	**Sensitivity, % (95% CI)**	**Specificity, % (95% CI)**	**AUC**
Presence of blood (WHO guideline^7^)	*Shigella* diarrhoea	315 (4·7)	14·5 (12·1–17·3)	96·5 (96·0–97·0)	0·555
*Shigella* score	*Shigella* diarrhoea	1322 (19·8)	50·4 (46·7–54·1)	84·0 (83·0–84·9)	0·783
Viral score	Viral diarrhoea	1462 (21·9)	33·9 (31·8–36·1)	82·9 (81·8–84·0)	0·659
*Cryptosporidium* score	*Cryptosporidium* diarrhoea	957 (14·3)	36·1 (27·5–45·4)	86·1 (85·2–86·9)	0·701

Analysis included all episodes of diarrhoea with complete valid quantitative PCR results and clinical characteristics for the top ten pathogens (n=6676). AUC=area under the curve of the receiver operating characteristic curve.

Overall, 3142 (47·1%) of 6676 diarrhoeal episodes in the MAL-ED observational cohort study were treated with an antibiotic. Defining appropriate antibiotic treatment post hoc, as treatment of *Shigella* diarrhoea with either a macrolide or a fluoroquinolone, only 260 (8·3%) of all 3142 antibiotic courses for diarrhoea were appropriate (table 4). Of 736 *Shigella* diarrhoea episodes, 260 (35·3%) were treated appropriately, 190 (25·8%) were treated inappropriately, and 286 (38·9%) were not treated. Taken together, this translates to one appropriately treated episode of *Shigella* diarrhoea for every 12·2 diarrhoea episodes that were treated inappropriately. Adherence to existing WHO guidelines would greatly restrict antibiotic use, in that only 315 (4·7%) of 6676 bloody diarrhoea episodes would be treated. However, only 107 (14·5%) of 736 *Shigella* episodes would be treated, with one appropriately treated episode for every 7·8 episodes treated inappropriately. Application of the *Shigella* score would treat about half of all *Shigella* episodes, while improving the efficiency of antibiotic use, with one appropriate episode treated for every 3·5 episodes treated inappropriately.

**Table 4:** Potential impact of adherence to current guidelines or the model-based prediction score on appropriate antibiotic use for diarrhoea

	***Shigella* diarrhoea (n=736)**			**Non-*Shigella* diarrhoea (n=5940)**		Ratio of appropriate to either inappropriate treatment or non-treatment*
	Appropriate antibiotics	Inappropriate antibiotics	Inappropriately not treated	Inappropriate antibiotics	Not treated	
Observed in this study	260 (35·3%)	190 (25·8%)	286 (38·9%)	2692 (45·3%)	3248 (54·7%)	260:3168 (1:12·2)
Presence of blood (WHO guideline)	107 (14·5%)	NA	629 (85·5%)	208 (3·5%)	5732 (96·5%)	107:837 (1:7·8)
*Shigella c*linical score	371 (50·4%)	NA	365 (49·6%)	951 (16·0%)	4989 (84·0%)	371:1316 (1:3·5)

Data are n (%), unless otherwise specified. Analysis includes all episodes of diarrhoea with complete valid quantitative PCR results and clinical characteristics for the top ten pathogens (n=6676). NA=not applicable.

* Numbers in parentheses represent the ratio of appropriate to either inappropriate treatment or non-treatment per appropriate treatment.

## Discussion

In this study, the application of quantitative molecular diagnostics substantially altered previous estimates of diarrhoea aetiology obtained from the prospective, multisite MAL-ED cohort study. Of the five pathogens with highest overall attributable incidence of diarrhoea, only rotavirus and enterotoxigenic *E coli*, were in the top five using the original microbiological work-up.^14^ Although underattribution to *Shigella* by traditional bacteriology has been previously documented,^6^ underattribution was substantially higher in this study: quantitative PCR showed that the *Shigella* burden was more than five times higher than the burden estimated by culture. In the second year of life, more than one of every five attributable episodes was *Shigella* diarrhoea. Although evidence^6^ also suggests that the burden of adenovirus 40/41 has been substantially underestimated, sapovirus had the second highest overall attributable incidence of diarrhoea in these diverse community settings, which is notable. A high prevalence of sapovirus diarrhoea has been reported previously,^25^ but not across such diverse geographical locations. The burden of astrovirus diarrhoea was also higher than previously estimated. Among the ten pathogens that were responsible for the majority of cases of infectious diarrhoea, considerable heterogeneity was observed in the pathogen hierarchy by site. In comparison to the Global Enteric Multicenter Study,^6^ in which a similar quantitative PCR analysis was done, this cohort study captured the full denominator of diarrhoea severity in the community setting. However, four of the top five aetiologies (*Shigella*, rotavirus, adenovirus 40/41, and enterotoxigenic *E coli*) were shared. The modelled associations between pathogen quantity and diarrhoea were largely consistent between the two studies (appendix).

In this re-analysis of the MAL-ED community-based cohort study with active surveillance for diarrhoea, nearly two-thirds of diarrhoea could be attributed to a pathogen by quantitative PCR, with a large increase in attribution in the second year of life. We have previously shown that quantitative PCR further narrowed this aetiologic gap for severe diarrhoea,^6^ suggesting that a sizeable proportion of diarrhoea collected in this study in the first year of life might be non-infectious. Although we used a standardised diarrhoea definition of three or more loose stools in a 24 h period, this definition can lack specificity in young children and those who are exclusively breastfed.^26^

Diarrhoea is a syndrome with many causes, and the clinical determination of aetiology on the basis of the diarrhoea episode is difficult. We identified some phenotypes of aetiology-specific diarrhoea, with rotavirus and *Shigella* most strongly associated with specific characteristics. In comparison, enterotoxigenic *E coli* episodes were poorly differentiated, thus a tailored clinical case definition for interventional studies might be difficult.^27^ Despite using a stringent metric to identify aetiology for individual episodes, we found multiple aetiologies in one of every five episodes in which an infectious aetiology was found. The presence of copathogens predictably altered the clinical phenotype, suggesting that interventional studies that do not account for co-pathogens in pathogen-specific case definitions might bias efficacy estimates towards the null.

In this study, we found that blood in stool is a poor marker for shigellosis, which is consistent with the conclusions of previous studies.^6^, ^9^, ^10^ *Shigella* has been associated with mortality in similar resource-limited settings, an association that extends beyond dysentery,^10^ and in the companion Article by Rogawski and colleagues^28^ *Shigella* infection was strongly associated with linear growth deficits. Thus, it is possible that current indications for antibiotic treatment of diarrhoea are too restrictive.^7^, ^10^ A previous clinical trial^29^ found that watery *Shigella* responds to treatment, and a randomised controlled trial (NCT03130114) of antibiotic therapy for non-dysenteric diarrhoea in high-risk children is ongoing. If treatment of non-dysenteric *Shigella* is found to be beneficial, in the absence of point-of-care testing, an improved syndromic approach could be of benefit. In this study, in addition to blood in stool, incorporation of other clinical characteristics stratified by age improved identification of shigellosis.

We have previously reported antibiotic use at these eight sites (4·9 courses per child-year),^8^ but a substantial proportion of cases did not seem to be clinically indicated. Almost half of all diarrhoea episodes in this study were treated with antibiotics, and based on a quantitative PCR gold standard, 91·7% of these antibiotic courses were inappropriate. Since almost 1 billion episodes of diarrhoea occur annually in children aged 5 years or younger,^1^ this translates to a substantial amount of inappropriate antibiotic exposure, which is high even in comparison to the amount of inappropriate antibiotic prescribed in developed countries for respiratory infections.^30^ Better adherence to guidelines is clearly needed,^8^ however the guidelines could also be revisited in view of improved data on diarrhoeal aetiology. We provide proof of concept that the use of readily-available criteria could reduce undertreatment of shigellosis with a modest trade-off of increased inappropriate antibiotic treatment. However, an accurate point-of-care diagnostic could offer substantial additional benefit. Optimisation of antimicrobial use is a strategic objective of the WHO Global Action Plan on antimicrobial resistance, and we agree with calls^31^ that guidelines should consider inappropriate antibiotic use during their development process.

This study has several limitations. First, the MAL-ED cohort study was designed to investigate long-term sequelae of early enteric infections, and thus was not powered for diarrhoea aetiology at the level of the site, and diarrhoeal severity was lower than studies done in the health-care setting. Application of these diagnostics in broader surveillance networks can help extend these estimates.^1^, ^5^ We identified a score for shigellosis to show that the syndromic treatment of diarrhoea could be improved, however any score for clinical use would need prospective derivation and validation, ideally in a study that can also assess the efficacy of such an approach. Our score is derived using the complete clinical phenotype of each diarrhoea episode, whereas less information will be available at the point of care, and early case detection and management in the setting of active surveillance might have changed the natural history of diarrhoea in patients that would otherwise have presented for care. Our definition of appropriate antibiotic use assumes a benefit for treatment of non-dysenteric *Shigella* and no benefit for treatment of other bacterial causes, and trials of expanded antibiotic treatment for diarrhoea are urgently needed to test this assumption. Without such data, modification of the current clinical algorithm is premature. Futhermore, our definition of appropriate antibiotic use takes into account local susceptibility information, but the evolution of antimicrobial resistance could alter this.

In summary, using quantitative molecular diagnostics we were able to identify ten pathogens responsible for the majority of community-based childhood infectious diarrhoea in diverse low-resource settings, including several pathogens for which burdens have been previously substantially underestimated. Although most cases of infectious diarrhoea were of viral aetiology, *Shigella* had the single highest attributable incidence. However, less than one in five episodes of shigellosis were accompanied by blood in stool. If non-dysenteric *Shigella* requires antibiotic therapy, it might be possible to leverage clinical characteristics to improve guidelines for the syndromic management of childhood diarrhoea in these settings.
