# Supplementary material for: Use of quantitative molecular diagnostic methods to assess the aetiology, burden, and clinical characteristics of diarrhoea in children in low-resource settings: a reanalysis of the MAL-ED cohort study
Source: Lancet Glob Health. 2018 Oct 1;6(12):e1309–18. doi: 10.1016/S2214-109X(18)30349-8 (PMC6227251; doi:10.1016/S2214-109X(18)30349-8)
Supplement: Supplementary appendix [file mmc1.pdf]

THE LANCET
Global Health

Supplementary appendix

This appendix formed part of the original submission and has been peer reviewed. We post it as supplied by the authors.

Supplement to: Platts-Mills JA, Liu J, Rogawski ET, et al, and The MAL-ED Network Investigators. Use of quantitative molecular diagnostic methods to assess the aetiology, burden, and clinical characteristics of diarrhoea in children in low-resource settings: a reanalysis of the MAL-ED cohort study. *Lancet Glob Health* 2018; published online Oct 1.http://dx.doi.org/10.1016/S2214-109X(18)30349-8.

Supplementary material

Supplementary Methods 3

Supplementary Results........ 4

Table S1. Real time PCR assays on TaqMan Array Card used for MALED diarrhoea aetiology analysis........ 4

Table S2. Attributable incidence of diarrhoea by quantitative PCR in MAL-ED by site and overall. 5

Table S3. Overall attributable fraction of diarrhoea and severe subsets by quantitative PCR in MAL-ED 6

Table S4. Quantitative aetiologic cut-offs for each pathogen – comparison between MAL-ED and the Global Enteric Multicenter Study (GEMS). 7

Table S5. Model-based prediction of aetiology: components, AUC, and cut-offs. 8

Table S6. Performance of overall model-based prediction of *Shigella* by site. 8

Table S7. Model-based prediction scores for individual non-*Shigella* bacteria and viruses. 8

Figure S1. Diarrhoea surveillance, stool sample collection, and stool testing and validity by qPCR. 9

Figure S2. Pathogen detection by qPCR in diarrhoeal stools. 10

Figure S3. Pathogen detection by the original microbiologic work-up in diarrhoeal stools from children that were included (n=1715, orange) or excluded (n=420, blue) from the qPCR re-analysis. 11

Figure S4. Pathogen detection by qPCR in diarrhoeal and non-diarrhoeal stools by age and site. 12

Figure S5. Relationship between pathogen quantity and diarrhoea. 14

Figure S6. Relationship between pathogen quantity and diarrhoea in children 0-11 months (blue) and 12-24 months of age (red). 15

Figure S7. Relationship between pathogen quantity and both severe (red) and non-severe (blue) diarrhoea. 16

Figure S8. Relationship between pathogen quantity and diarrhoea in MAL-ED (blue) and GEMS (red). 17

Figure S9. Comparison between estimates of overall attributable incidence by quantitative PCR vs. the original study microbiology. 18

Figure S10. Comparison between estimates of overall attributable incidence by quantification cycle vs copy number...... 19

Figure S11. Overall pathogen-specific incidence by attributable fraction method vs. aetiologic detections. 20

Figure S12. Overall attributable incidence for increasingly restrictive definitions of a non-diarrhoeal stool. 21

Figure S13. Attributable incidence of diarrhoea with specific clinical characteristics. 22

Figure S14. Diarrhoea episodes tested and with primary aetiology identified. 23

Figure S15. Model-based prediction of aetiology. For each disease, the left plot shows the score distribution by 3-month age intervals. All scores above the blue line, which 24

References........ 25

Additional MAL-ED Investigators

Angel Mendez Acosta[1]
Rosa Rios de Burga[1]
Cesar Banda Chavez[1]
Julian Torres Flores[1]
Maribel Paredes Olotegui[1]
Silvia Rengifo Pinedo[1]
Dixner Rengifo Trigoso[1]
Angel Orbe Vasquez[1]
Imran Ahmed[2]
Didar Alam[2]
Asad Ali[2]
Muneera Rasheed[2]
Sajid Soofi[2]
Ali Turab[2]
Aisha Yousafzai[2]
Anita KM Zaidi[2]
Binob Shrestha[3]
Bishnu Bahadur Rayamajhi[3]
Tor Strand[3,19]
Geetha Ammu[4]
Sudhir Babji[4]
Anuradha Bose[4]
Ajila T George[4]
Dinesh Hariraju[4]
M. Steffi Jennifer[4]
Sushil John[4]
Shiny Kaki[4]
Priyadarshani Karunakaran[4]
Beena Koshy[4]
Robin P Lazarus[4]
Jayaprakash Muliyil[4]
Preethi Ragasudha[4]
Mohan Venkata Raghava[4]
Sophy Raju[4]
Anup Ramachandran[4]
Rakhi Ramadas[4]
Karthikeyan Ramanujam[4]
Anuradha Rose[4]
Reeba Roshan[4]
Srujan L Sharma[4]
Shanmuga Sundaram E[4]
Rahul J Thomas[4]
William K Pan[5,6]
Ramya Ambikapathi[6,15]
J Daniel Carreon[6]
Viyada Doan[6]
Christel Hoest[6]
Stacey Knobler[6]
Mark A Miller[6]
Stephanie Psaki[6]
Zeba Rasmussen[6]
Stephanie A Richard[6]
Karen H Tountas[7]
Erling Svensen[8,9]
Caroline Amour[9]
Eliwaza Bayyo[9]
Regisiana Mvungi[9]
John Pascal[9]
Ladislaus Yarrot[9]
Leah Barrett[10]
Rebecca Dillingham[10]
William A Petri, Jr[10]
Rebecca Scharf[10]
AM Shamsir Ahmed[11]
Md Ashraful Alam[11]
Umma Haque[11]
Md Iqbal Hossain[11]
Munirul Islam[11]
Mustafa Mahfuz[11]
Dinesh Mondal[11]
Baitun Nahar[11]
Fahmida Tofail[11]
Ram Krishna Chandyo[12]
Prakash Sunder Shrestha[12]
Rita Shrestha[12]
Manjeswori Ulak[12]
Aubrey Bauck[13]
Robert Black[13]
Laura Caulfield[13]
William Checkley[13,6]
Gwenyth Lee[13,21]
Kerry Schulze[13]
Samuel Scott[14]
Laura E. Murray-Kolb[16]
A Catharine Ross[16]
Barbara Schaefer[16,6]
Suzanne Simons[16]
Laura Pendergast[17]
Cláudia B Abreu[18]
Hilda Costa[18]
Alessandra Di Moura[18]
José Quirino Filho[18,7]
Álvaro M Leite[18]
Noélia L Lima[18]
Ila F Lima[18]
Bruna LL Maciel[18]
Pedro HQS Medeiros[18]
Milena Moraes[18]
Francisco S Mota[18]
Reinaldo B Oriá[18]
Josiane Quetz[18]
Alberto M Soares[18]
Rosa MS Mota[18]
Crystal L Patil[20]
Cloupas Mahopo[22]
Angelina Maphula[22]
Emanuel Nyathi[22]

[1]A.B. PRISMA, Iquitos, Peru
[2]Aga Khan University, Karachi, Pakistan
[3]Walter Reed/AFRIMS Research Unit, Kathmandu, Nepal
[4]Christian Medical College, Vellore, India
[5]Duke University, Durham, NC, USA
[6]Fogarty International Center/National Institutes of Health, Bethesda, MD, USA
[7]Foundation for the National Institutes of Health, Bethesda, MD, USA
[8]Haukeland University Hospital, Bergen, Norway
[9]Haydom Lutheran Hospital, Haydom, Tanzania
[10]University of Virginia, Charlottesville, VA, USA
[11]icddr,b, Dhaka, Bangladesh
[12]Institute of Medicine, Tribhuvan University, Kathmandu, Nepal
[13]Johns Hopkins University, Baltimore, MD, USA
[14]Poverty Health and Nutrition Division, International Food Policy Research Institute, Washington DC, USA
[15]Purdue University, Department of Nutrition Science, IN, USA
[16]The Pennsylvania State University, University Park, PA, USA
[17]Temple University, Philadelphia, PA, USA
[18]Universidade Federal do Ceara, Fortaleza, Brazil
[19]University of Bergen, Norway
[20]University of Illinois at Chicago, IL, USA
[21]University of Michigan, Department of Epidemiology, MI, USA
[22]University of Venda, Thohoyandou, South Africa

Supplementary Methods

Nucleic acid extraction, qPCR setup, data quality

Nucleic acid was extracted with the QIAamp Fast DNA Stool mini kit (Qiagen, Hilden, Germany) with pretreatment steps that included bead beating. AgPath One Step RT-PCR reagents were used for qPCR reactions, which were performed on ViiA 7, or QuantStudio 7, or QuantStudio 12K Flex systems. Quantification cycles (Cqs) are the PCR cycle values at which fluorescence from amplification exceeds the background, which acts as an inverse metric of quantity of nucleic acid. Detections with a Cq less than or equal to 35 were positive. Valid results required proper functioning of controls: negative results for a sample were valid only when its external control MS2 was positive; positive results for a sample were valid only when the corresponding extraction blank was negative for the target; and we excluded data flagged by the real time PCR software, i.e. BADROX in combination with NOISE or SPIKE.

Sensitivity analyses for association between pathogen quantity and diarrhoea

To visualize the association between pathogen quantity and diarrhoea, we fit models that excluded the interaction between pathogen and age group and then generated model-predicted odds ratios between the quantities of each pathogen and a new dataset in which the quantity of that pathogen was set to 0, conditional on all other variables in the model (Figure S4). To determine the consistency of these associations, we compared the model-predicted odds ratios from the primary analysis to those between diarrhoea in the first and second year of life (Figure S5), between severe and non-severe diarrhoea (Figure S6), and from a prior study in which this approach was used (Figure S7).[1]

Comparison of estimates with original microbiology

To compare attributable incidence estimates between qPCR and the original microbiology, we fit the same models but using dichotomous exposures for the original microbiologic work-up. The model was otherwise specified identically, and the calculation of the AFes, attributable incidence, and confidence intervals was also the same (Figure S8).

Derivation of pathogen target copy numbers from quantification cycle (Cq) values

For derivation of pathogen target copy numbers from Cqs, we generated standard curves every six months at each laboratory using combined positive control materials of known copy number (constructed plasmids for DNA targets and in vitro transcripts for RNA targets). The combined extraction and amplification efficiencies were calculated as external control MS2 copy numbers in the sample divided by input MS2 copy numbers (defined as 95% percentile of MS2 signal in all the clinical samples by site to avoid the variation in reagent concentrations among sites). Target copy numbers were then calculated from Cq values and adjusted for the extraction and amplification efficiencies of the specimen from the external control. To avoid over-adjustment of pathogen quantity, the efficiency was forced to be > 0.1%. These values were then used in place of the pathogen quantification cycle values to generate attributable incidence estimates (Figure S9).

Identification of pathogen-specific quantitative cut-offs for aetiology

To identify model-derived Cq cutoffs for diarrhoea-associated pathogen, we fit models that excluded the interaction between pathogen and age group but were otherwise identical to those used for the AF calculations. The aetiologic cut-off was then defined as the quantification cycle at which the point estimate of the odds ratio was greater than or equal to 2, and thus the AFe was greater than or equal to 0.5 (Table S2 and Figure S4; detections above the aetiologic cut-off are shown in blue for each pathogen for which a cut-off could be identified). Cut-off values were also converted to the copy number equivalent (Table S2). Incidence estimates based on these cut-offs were compared to the population attributable incidence estimates (Figure S10). The prevalence of diarrhoeal stools with a primary aetiology was identified by month and by site (Figure S13).

Sensitivity analyses for the definition of a non-diarrhoeal stool

For the primary analysis, non-diarrhoeal stools were required to be collected at least 7 days both before and after any surveillance-identified day of diarrhoea. To compare progressively more restrictive definitions of a non-diarrhoeal stool, we subset non-diarrhoeal stools to those that were 14 and 28 days removed from diarrhoea. All diarrhoeal stools were retained. We then re-generated attributable incidence estimates for these subsets (Figure S11).

Attributable incidence estimates for subgroups with and without specific clinical characteristics

To calculate the attributable incidence of diarrhoea with and without specific clinical characteristics, the same model used in the overall analysis was fit, and model-based odds ratios were used to calculate AFes. Then, AFes were summed for each subset and attributable incidence was calculated based on the surveilled incidence of that subset of diarrhoea (Figure S12).

Validation of model-based scores by site

For each pathogen or pathogen category in the main analysis, the score components, distributions by 3-month age intervals as well as the ROC plots are shown in Table S4 and Figure S14. To validate the *Shigella* score by site, we performed an ROC analysis for each site using the same score and score cut-off (Table S5).

Supplementary Results

Table S1. Real time PCR assays on TaqMan Array Card used for MALED diarrhoea aetiology analysis.

	Pathogen	Gene	Included in diarrhoea aetiology analysis	Assay in the original microbiologic work-up*
Viruses	Adenovirus 40/41	Fiber gene	✓	EIA†
	Astrovirus	Capsid	✓	EIA
	Norovirus GI/GII	GI ORF1-2 and GII ORF1-2	✓	PCR§
	Rotavirus	*NSP3*	✓	EIA
	Sapovirus	*RdRp*	✓	Not tested
Bacteria	EAEC**	*aaiC, aatA*	✓	Culture/PCR
	Atypical EPEC**	*eae*	✓	Culture/PCR
	Typical EPEC**	*bfpA*	✓	Culture/PCR
	ETEC**	*LT, STh* and *STp*	✓	Culture/PCR
	STEC**	*stx1, stx2*	✓	Culture/PCR
	Aeromonas	Aerolysin	✓	Culture
	Campylobacter spp	*cadF (C. jejuni/coli)* and *cpn60* (*Campylobacter* spp)	✓ (*C. jejuni/coli*)	EIA††
	Helicobacter pylori	*ureC*	✓	Not tested
	Plesiomonas shigelloides	*gyrB*	✓	Culture
	Salmonella	*ttr*	✓	Culture
	Shigella/EIEC	*ipaH*	✓	Culture
	Vibrio cholerae	*hlyA*	✓	Culture
Fungi	*Enterocytozoon bieneusi*	*ITS*	✓	Not tested
	Encephalitozoon intestinalis	SSU rRNA	✓	Not tested
Protozoa	*Cryptosporidium*	18S rRNA	✓	EIA
	Cyclospora cayetanensis	18S rRNA	✓	Microscopy
	Cystoisospora belli	18S rRNA	✓	Microscopy
	Entamoeba histolytica	18S rRNA	✓	EIA
	Giardia	18S rRNA	✓	EIA
Helminth	*Ancylostoma duodenale*	*ITS2*	✓	Microscopy
	Ascaris lumbricoides	*ITS1*		Microscopy
	Necator americanus	*ITS2*		Microscopy
	Strongyloides stercoralis	Dispersed repetitive sequence	✓	Microscopy
	Trichuris trichiura	18S rRNA	✓	Microscopy
Controls	MS2	*MS2g1*	N/A	N/A
	PhHV	*gB*	N/A	N/A

All the assays have been described previously and extensively validated.[1-3] * Conventional methods were conducted in non-diarrhoeal stools monthly in the first year of life, but only quarterly in the second year of life (qPCR was conducted in non-diarrhoeal stools monthly for both years). †Pan-adenovirus EIA only. §Norovirus PCR performed on all diarrhoeal stools as well as all non-diarrhoeal stools from a randomly selected 10% of participants. ***E. coli* pathotypes were defined as follows: EAEC (*aaiC*, or *aatA*, or both), atypical EPEC (*eae* without *bfpA*, *stx1*, and *stx2*), typical EPEC (*bfpA*), ETEC (STh, STp, or LT), STEC (*eae* without *bfpA* and with *stx1*, *stx2*, or both). ††A single EIA was used, which has been shown to detect some *Campylobacter* species other than *C. jejuni* and *C. coli*.[4]

Table S2. Attributable incidence of diarrhoea by quantitative PCR in MAL-ED by site and overall.

	Dhaka, Bangladesh	Vellore, India	Bhaktapur, Nepal	Naushero Feroze, Pakistan	Venda, South Africa*	Haydom, Tanzania	Fortaleza, Brazil*	Loreto, Peru*	Overall
Overall incidence	361·9	211·5	233·5	632·1	62·2	128·5	50·9	449	273·8
Shigella	65·2 (57·4–76·9)	27·9 (21·0–35·8)	18·6 (14·7–24·1)	33·6 (26·3–44·4)	4·2 (1·5–8·1)	9·3 (1·3–17·8)	6·8 (3·4–11·5)	42·3 (32·5–53·0)	26·1 (23·8–29·9)
Sapovirus	28·6 (17·9–39·2)	20·3 (13·5–27·0)	23·2 (16·8–29·1)	36·6 (20·4–54·2)	3·5 (1·0–6·0)	11·2 (4·9–18·5)	3·7 (1·4–6·6)	54·0 (43·3–67·0)	22·8 (18·9–27·5)
Rotavirus	57·6 (50·4–66·3)	19·5 (14·8–26·1)	23·3 (19·3–27·8)	25·9 (19·7–32·4)	2·5 (0·5–4·9)	13·4 (7·6–21·1)	1·5 (0·0–3·3)	19·5 (13·9–25·3)	20·7 (18·8–23·0)
Adenovirus 40/41	86·5 (73·8–105·6)	12·8 (6·0–19·9)	4·3 (0·7–8·6)	7·3 (0·0–18·8)	2·3 (0·1–4·8)	7·1 (2·0–13·5)	3·0 (0·6–6·0)	32·4 (23·1–44·4)	19·0 (16·8–23·0)
ETEC	55·5 (44·2–68·7)	17·3 (12·7–25·4)	15·2 (10·4–20·7)	17·6 (10·7–31·0)	1·9 (0·4–4·3)	22·5 (13·1–36·5)	2·3 (0·7–5·2)	16·8 (9·7–30·3)	18·8 (16·5–23·8)
Norovirus	16·4 (9·7–26·3)	7·3 (2·4–12·3)	16·5 (12·3–22·7)	16·1 (7·4–32·9)	3·7 (1·5–8·5)	14·2 (7·3–23·8)	6·3 (3·0–10·4)	44·8 (35·2–61·2)	15·4 (13·5–20·1)
Astrovirus	18·8 (8·3–28·9)	13·7 (8·6–21·2)	9·2 (5·8–13·1)	28·7 (18·2–43·9)	2·8 (0·8–6·0)	4·5 (0·8–9·1)	1·6 (0·4–3·7)	39·5 (31·3–53·6)	15·0 (12·0–19·5)
C. jejuni/coli	14·2 (3·8–29·8)	11·1 (5·1–17·2)	8·2 (2·1–15·7)	14·1 (1·3–31·1)	3·2 (0·5–6·6)	6·9 (0·0–17·9)	2·4 (0·5–6·6)	37·6 (24·5–51·9)	12·1 (8·5–17·2)
Cryptosporidium	6·9 (2·3–11·8)	5·6 (2·7–12·4)	4·1 (1·5–6·5)	8·8 (4·0–18·6)	0·2 (0·0–1·2)	2·2 (0·0–7·1)	0·3 (0·0–1·1)	18·2 (10·8–26·7)	5·8 (4·3–8·3)
tEPEC	4·6 (0·2–11·4)	5·9 (1·0–12·7)	3·7 (1·0–7·5)	15·6 (2·0–34·8)	0·6 (00–3·2)	4·2 (0·1–11·4)	0·1 (0·0–0·9)	6·5 (0·8–13·3)	5·4 (2·8–9·3)
EAEC		0·2 (0·0–2·8)	0·4 (0·0–3·4)	2·7 (0·3–15·1)	2·2 (0·0–8·6)	9·7 (0·7–28·9)	4·0 (0·2–9·8)	1·4 (0·1–8·2)	2·5 (1·1–6·0)
Giardia	0·9 (0·0–5·5)	0·7 (0·0–4·2)	0·2 (0·0–1·5)					8·5 (2·1–18·8)	1·2 (0·4–2·8)
Plesiomonas	0·8 (0·0–6·5)	0·7 (0·0–4·6)	0·2 (0·0–0·8)			1·9 (0·0–7·3)		2·6 (0·0–9·2)	0·7 (0·1–2·2)
aEPEC	1·0 (0·1–2·4)	0·1 (0·0–0·5)	0·9 (0·1–3·5)					2·7 (0·5–7·6)	0·6 (0·1–1·5)
Isospora	0·3 (0·0–1·9)		0·2 (0·0–1·2)				0·9 (0·0–2·8)	2·9 (0·4–7·7)	0·5 (0·1–1·1)
V. cholerea	1·1 (0·2–2·6)	1·3 (0·2–2·8)							0·3 (0·1–0·6)
Cyclospora	0·9 (0·0–3·0)		0·6 (0·0–2·5)					0·9 (0·0–2·6)	0·3 (0·1–0·7)
E. bieneusi	0·3 (0·0–2·7)		0·1 (0·0–1·7)	0·3 (0·0–2·1)				1·7 (0·1–10·8)	0·3 (0·0–1·8)
E. histolytica	1·6 (0·3–3·5)	0·6 (0·0–1·7)							0·3 (0·1–0·5)
Salmonella		0·2 (0·0–1·5)			0·3 (0·0–1·7)	0·4 (0·0–1·7)	0·5 (0·0–1·5)		0·2 (0·0–0·7)
Strongyloides								1·4 (0·0–4·5)	0·2 (0·0–0·6)
Ancylostoma								1·4 (0·3–2·9)	0·2 (0·1–0·4)
H. pylori					0·1 (0·0–0·9)	0·4 (0·0–3·5)			0·1 (0·0–1·1)
Trichuris	0·2 (0·0–2·5)						0·1 (0·0–0·8)		0·0 (0·0–0·4)
E. intestinalis	0·1 (0·0–0·5)								0·0 (0·0–0·3)

Estimates are per 100 child-years (95% CIs) and are ordered by overall attributable incidence. All pathogens with a point estimate greater than 0 for at least one site or overall are shown. EAEC=enteroaggregative E. coli. aEPEC=atypical enteropathogenic E. coli. tEPEC=typical enteropathogenic E. coli. ETEC=enterotoxigenic E. coli

Table S3. Overall attributable fraction of diarrhoea and severe subsets by quantitative PCR in MAL-ED

Pathogen	Overall	Moderate-to-severe diarrhoea*	Severity score > 6†
Shigella	10·2; 9·1–11·4	17·6; 15·2–20·3	8·4; 6·6–10·5
Sapovirus	8·7; 7·2–10	9·2; 6·3–12·7	9·0; 6·1–11·7
Rotavirus	8·1; 6·9–9·5	11·8; 9·5–14·2	14·1; 11·9–16·0
Adenovirus 40/41	8; 7·1–8·9	8·3; 6·1–10·7	7·4; 5·5–9·5
ETEC	7; 6·1–8·5	8·4; 5·3–12·3	9·1; 6·3–12·4
Norovirus	5·8; 4·8–7·2	5·0; 3·4–8·2	6·5; 4·6–9·7
Astrovirus	5·7; 4·5–7·1	5·8; 3·1–8·8	5·4; 2·7–7·9
C. jejuni/coli	4·7; 3·2–6·4	7·0; 3·8–10·2	3·5; 1·0–6·5
Cryptosporidium	2·3; 1·6–3·1	3·0; 1·4–5·2	1·8; 1·0–3·7
Typical EPEC	1·8; 1–2·9	3·0; 1·2–6·0	3·6; 1·8–6·2

*As defined in the Global Enteric Multicenter Study.1 †Derived from the Vesikari score.[5]

Table S4. Quantitative aetiologic cut-offs for each pathogen – comparison between MAL-ED and the Global Enteric Multicenter Study (GEMS).

Pathogen	MAL-ED Cq (copy number)	GEMS[1] Cq
Adenovirus 40/41	24·0 (9·9 x 10^7 copies/g)	22·7
Sapovirus	26·1 (1·5 x 10^7 copies/g)	NA
Astrovirus	23·7 (1·9 x 10^9 copies/g)	22·2
C. jejuni/coli	21·8 (2·6 x 10^8 copies/g)	15·4
ST-ETEC	23·5 (1·3 x 10^8 copies/g)	22·8
Norovirus GII	27·2 (1·7 x 10^8 copies/g)	23·4
Shigella	28·8 (6·2 x 10^5 copies/g)	27·9
Typical EPEC	17·8 (1·7 x 10^9 copies/g)	16·0
Rotavirus	31·7 (1·9 x 10^6 copies/g)	32·6
Cryptosporidium	22·0 (3·3 x 10^8 copies/g)	24·0
Isospora	33·8 (5·8 x 10^3 copies/g)	NA
Strongyloides	30·4 (2·0 x 10^4 copies/g)	NA
Entamoeba histolytica	30·0 (9·4 x 10^6 copies/g)	32·8
Vibrio cholerae	32·0 (5·1 x 10^5 copies/g)	33·8
Helicobacter pylori	NA	30·8
Salmonella	NA	30·7
Cyclospora cayetanensis	NA	29·6

We defined aetiologic detections as all episode- and quantity-specific detections with an episode attributable fraction (AFe) ≥ 0.5. Here, cut-offs are shown for ST-ETEC and norovirus GII, but for the analysis of clinical characteristics and model-based prediction scores, a combined AFe ≥ 0·5 for ST- and LT-ETEC and norovirus GI and GII respectively was used to identify aetiologic detections. Pathogens are stratified by identification of an aetiologic cut-off in MAL-ED and then ordered by prevalence in diarrhoea. EPEC= enteropathogenic E. coli. ST-ETEC=heat-stabile toxin-producing enterotoxigenic E. coli.

Table S5. Model-based prediction of aetiology: components, AUC, and cut-offs.

	Model-based prediction score							
	Blood	Fever	Prolonged diarrhoea (≥ 7 days)	Dehydration	Vomiting	High frequency (> 6 loose stools in 24 hours)	Child age (0-3,4-6,7-9,10-12,13-15,16-18,19-21,22-24 months)	Cut-off with >80% specificity
Presence of blood (WHO guideline[6])*	1	N/A	N/A	N/A	N/A	N/A	N/A	≥ 1
Shigella score	6	1	2	0	-2	1	0, 3, 9, 12, 14, 15, 17, 17	≥ 17
Viral score	-4	0	-1	2	3	1	0, 3, 6, 7, 7, 5, 5, 4	≥ 8
Cryptosporidium score	-3	1	2	1	1	0	0, -2, 6, 7, 8, 8, 9, 11	≥ 11

Here, model-based prediction scores were determined and fit with a receiver operating characteristic curve as described in the methods. Each number represents the points assigned for each characteristics; for age, the eight numbers for each score represent the points assigned for child age for each of the eight 3-month intervals The lowest score with greater than 80% specificity is shown. Table 3 shows the associated test characteristics. *Presence of blood.[6] WHO=World Health Organization.

Table S6. Performance of overall model-based prediction of *Shigella* by site.

Site	# Episodes	# Shigella episodes (%)	Sensitivity (%)	Specificity (%)	Episodes with score above cut-off	AUC
Dhaka, Bangladesh	1379	271 (19·7)	45·8 (39·7–51·9)	84·7 (82·5–86·8)	21·2	0·769
Vellore, India	631	95 (15·1)	52·6 (42·1–63)	89·7 (86·9–92·2)	16·6	0·828
Bhaktapur, Nepal	904	79 (8·7)	72·2 (60·9–81·7)	83·4 (80·7–85·9)	21·5	0·859
Naushero Feroze, Pakistan	1815	101 (5·6)	41·6 (31·9–51·8)	84·5 (82·7–86·2)	16·9	0·756
Venda, South Africa	115	9 (7·8)	33·3 (7·5–70·1)	81·1 (72·4–88·1)	20·0	0·668
Haydom, Tanzania	157	13 (8·3)	0·0 (0·0–24·7)	93·8 (88·5–97·1)	5·7	0·680
Fortaleza, Brazil	90	11 (12·2)	27·3 (6·0–61·0)	73·4 (62·3–82·7)	26·7	0·631
Loreto, Peru	1585	157 (9·9)	58·6 (50·5–66·4)	80·7 (78·6–82·8)	23·2	0·804

Here, the same score derived from the overall data (see Methods, Table S3, and Table 3) was used to identify test characteristics for each site.

Table S7. Model-based prediction scores for individual non-*Shigella* bacteria and viruses.

Test	Disease	% Test positive	Sensitivity (%)	Specificity (%)	AUC
C. jejuni/coli score	*C. jejuni/coli* diarrhoea	1290 (19·3)	43·4 (35·6–51·5)	81·3 (80·3–82·2)	0·673
ETEC	ETEC diarrhoea	595 (8·9)	15·7 (12·5–19·4)	91·6 (90·9–92·3)	0·657
tEPEC	tEPEC diarrhoea	560 (8·4)	24·1 (10·3–43·5)	91·7 (91·0–92·3)	0.708
Adenovirus 40/41	Adenovirus 40/41 diarrhoea	937 (14·0)	17·6 (14·0–21·6)	86·2 (85·3–87·0)	0·573
Astrovirus	Astrovirus diarrhoea	1290 (19·3)	31·7 (26·5–37·2)	81·3 (80·3–82·2)	0·625
Norovirus	Norovirus diarrhoea	575 (8·6)	19·1 (14·9–24·0)	91·9 (91·2–92·5)	0·659
Rotavirus	Rotavirus diarrhoea	1316 (19·7)	40·4 (36·3–44·7)	82·2 (81·2–83·1)	0·680
Sapovirus	Sapovirus diarrhoea	829 (12·4)	23·9 (20·4–27·8)	88·6 (87·8–89·4)	0·693

Table S8. Performance of model-based prediction of diarrhoea aetiology in moderate-to-severe diarrhoea for *Shigella*

	Disease	Episodes positive (%)	Sensitivity (%)	Specificity (%)	AUC
Presence of blood (WHO guideline[6])	*Shigella* diarrhoea	315 (32·7)	66·0 (58·4–73·3)	74·0 (70·8–77·0)	0·700
Shigella score	*Shigella* diarrhoea	258 (26·8)	66·7 (58·8–73·9)	81·3 (78·4–83·9)	0·824

Analysis includes all episodes of diarrhoea with complete valid qPCR results and clinical characteristics for the top ten pathogens (n=963). AUC = area under the curve of the receiver operating characteristic (ROC) curve. WHO=World Health Organization.

Figure S1. Diarrhoea surveillance, stool sample collection, and stool testing and validity by qPCR.

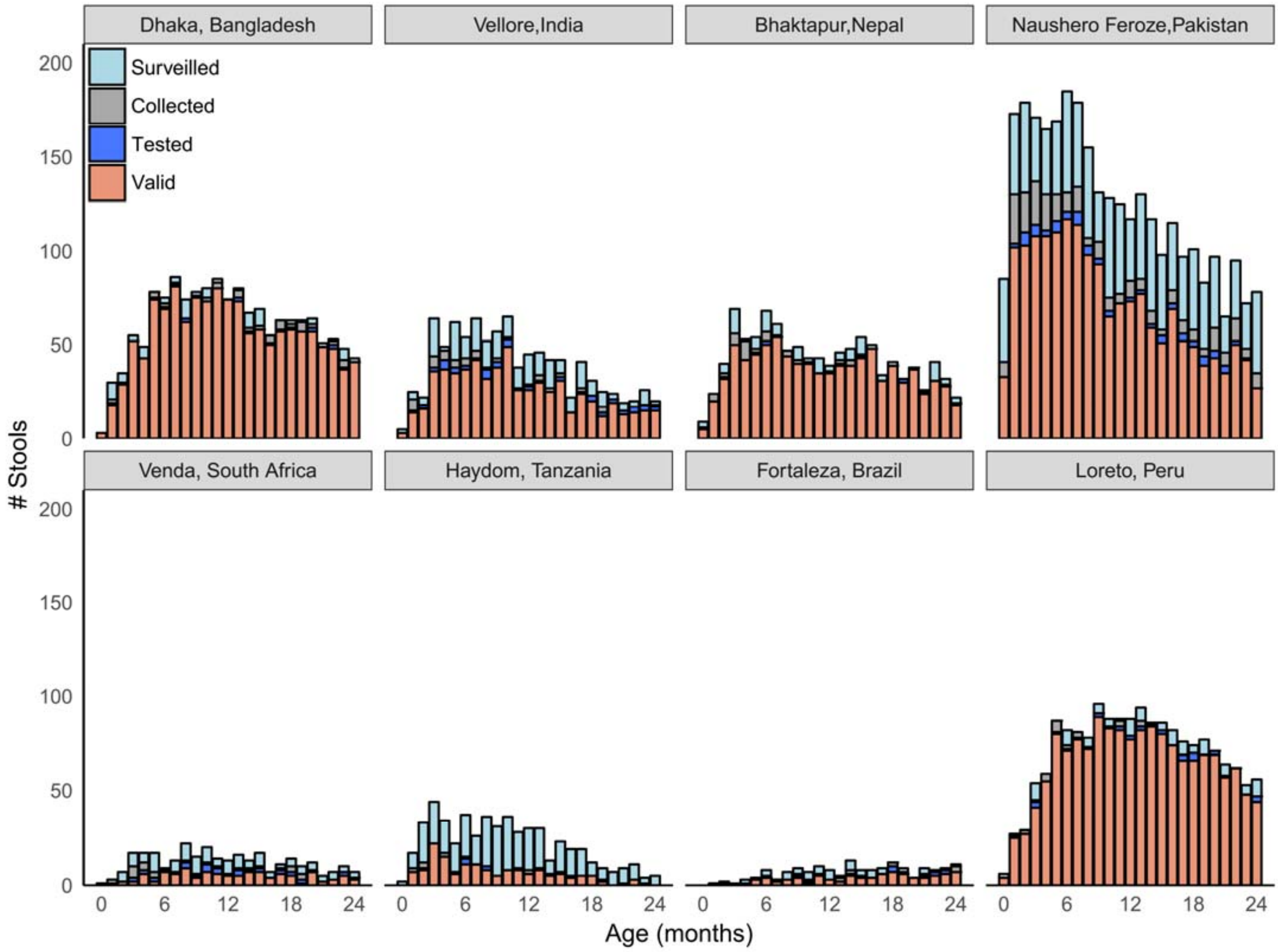

Figure S2. Pathogen detection by qPCR in diarrhoeal stools. All pathogens included in the aetiology analysis are shown. All detections with a quantification cycle < 35 are considered positive. EAEC=enteroaggregative E. coli. aEPEC=atypical enteropathogenic E. coli. tEPEC=typical enteropathogenic E. coli. ETEC=enterotoxigenic E. coli. STEC=Shiga-toxin producing E. coli.

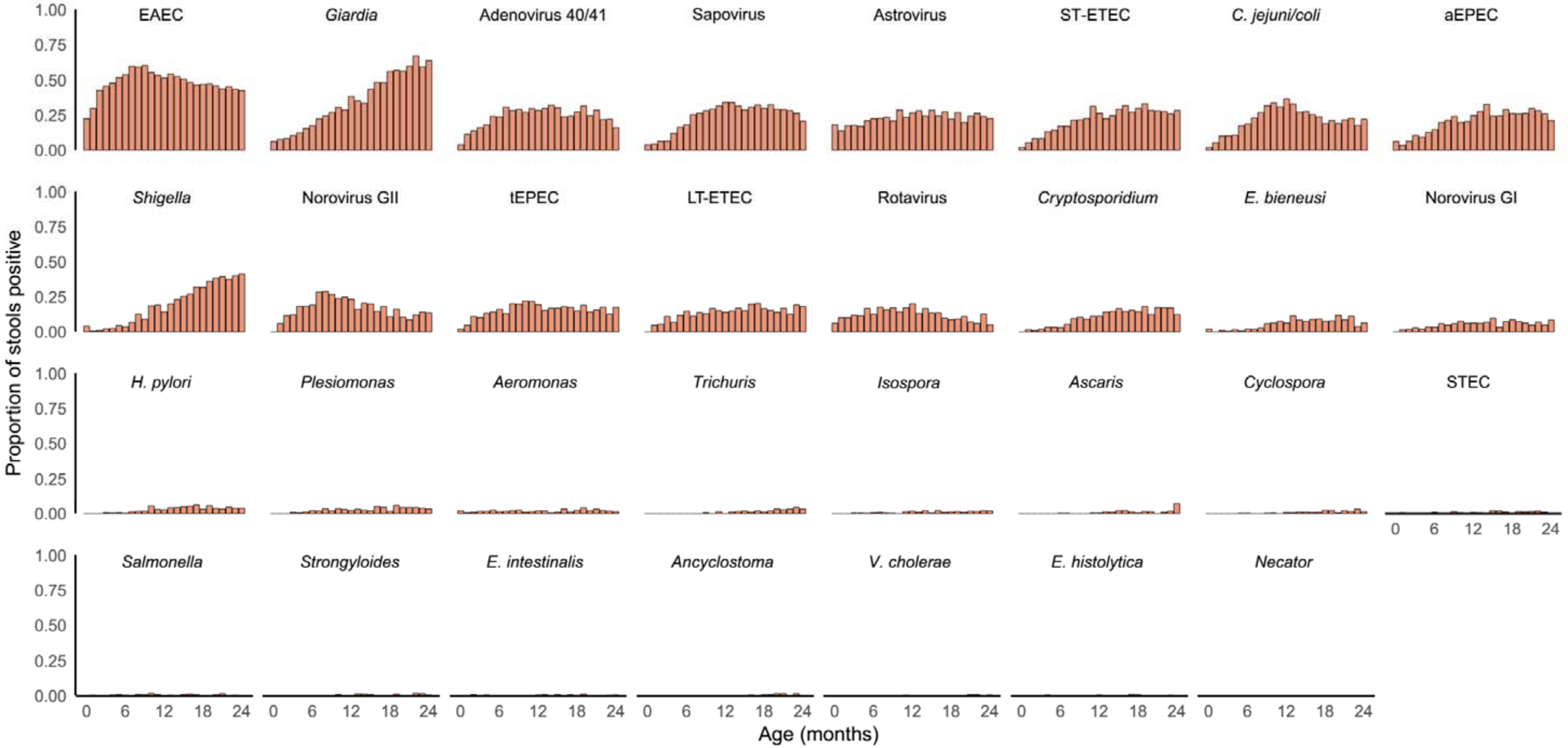

Figure S3. Pathogen detection by the original microbiologic work-up in diarrhoeal stools from children that were included (n=1715, orange) or excluded (n=420, purple) from the qPCR re-analysis. The top ten aetiologies of diarrhoea are shown, with the exception of sapovirus, which was not tested for in the original work-up. There were no statistically significant differences between pathogen detection in included and excluded children (from logistic regression model with outcome of pathogen detection and predictors of inclusion in the qPCR testing, age, and site). EPEC=typical enteropathogenic E. coli. ETEC=enterotoxigenic E. coli.

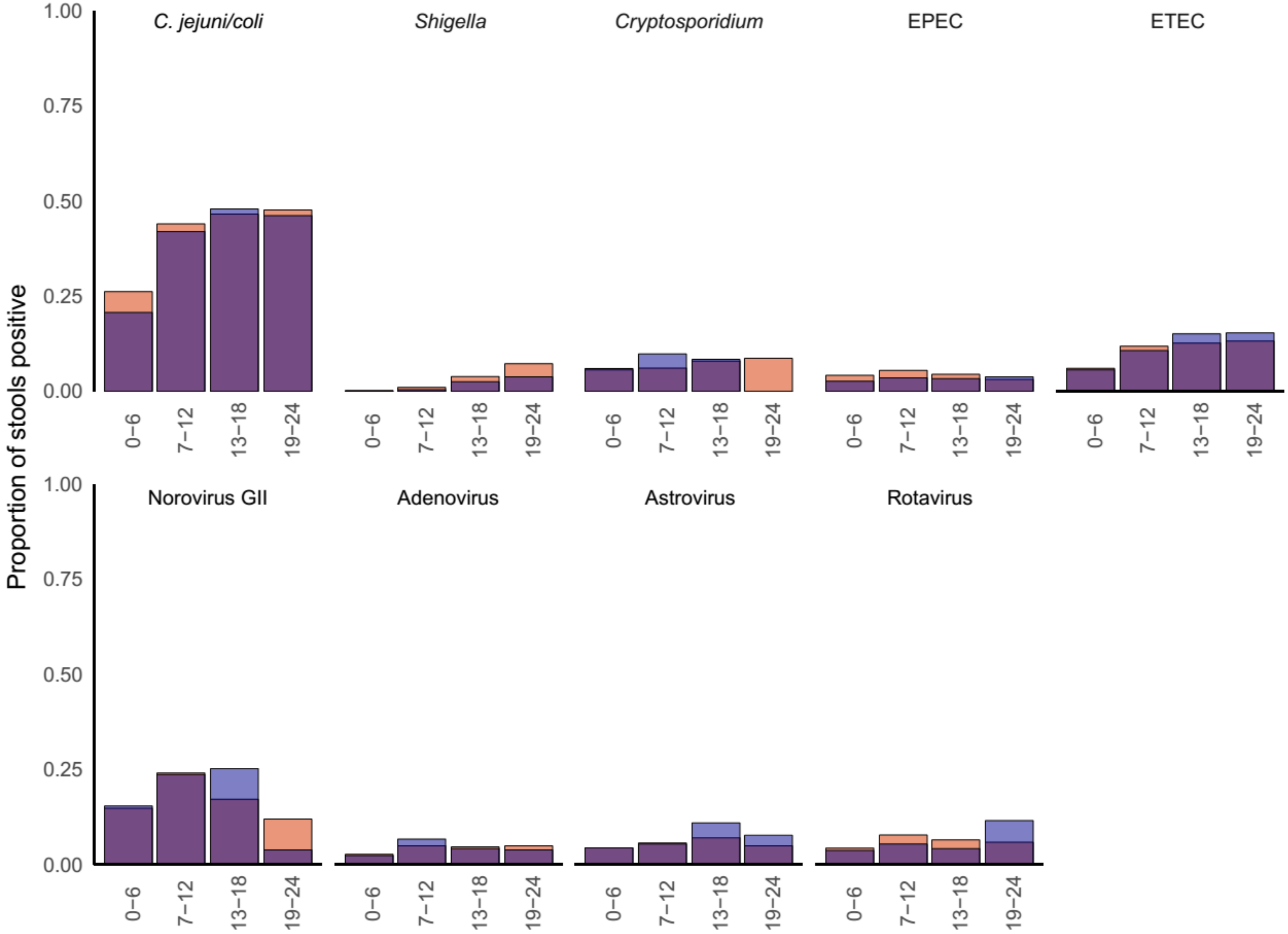

Figure S4. Pathogen detection by qPCR in diarrhoeal and non-diarrhoeal stools by age and site. The ten pathogens with highest overall attributable incidence are shown. All detections with a quantification cycle < 35 are considered positive. tEPEC=typical enteropathogenic E. coli. ETEC=enterotoxigenic E. coli.

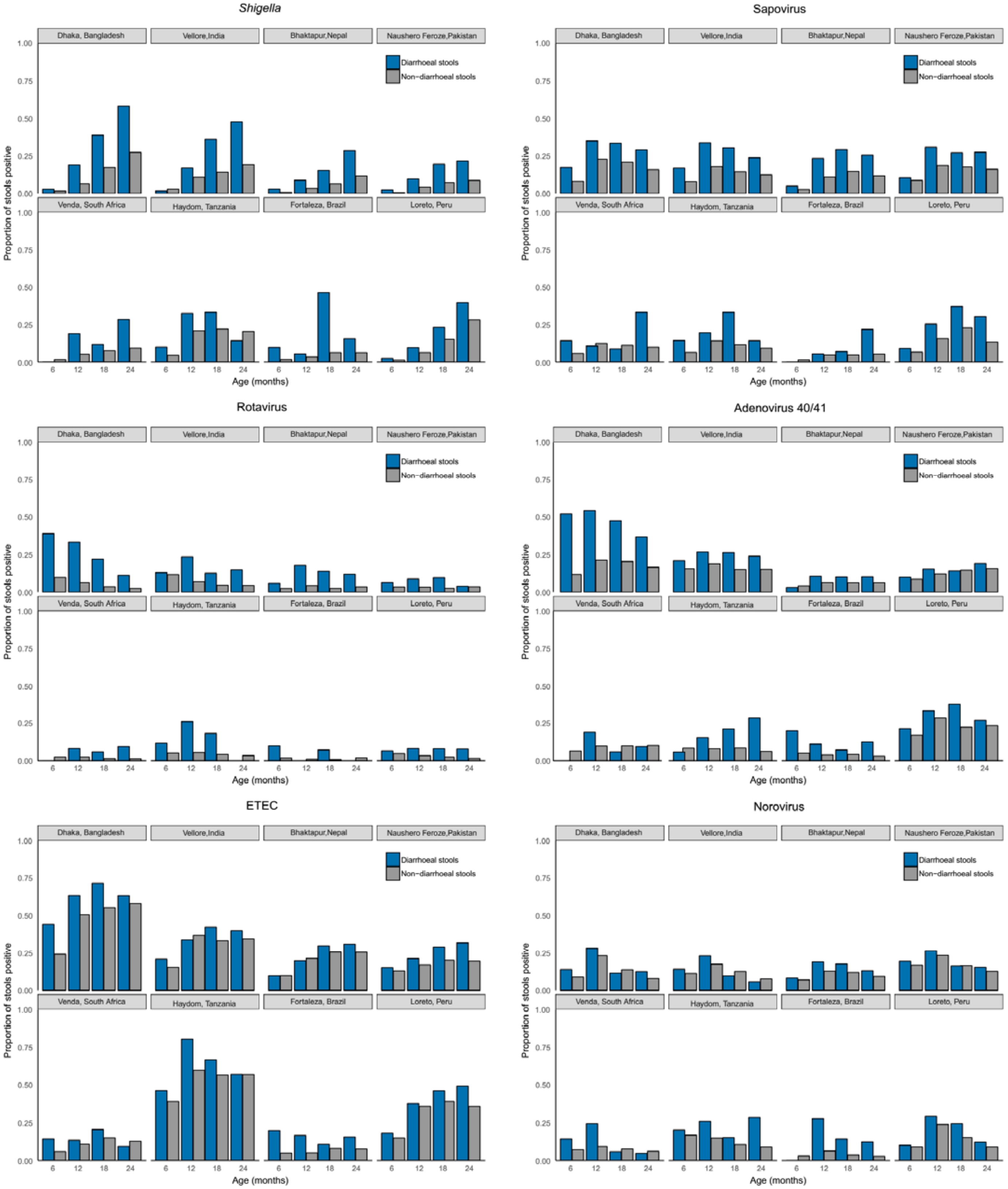

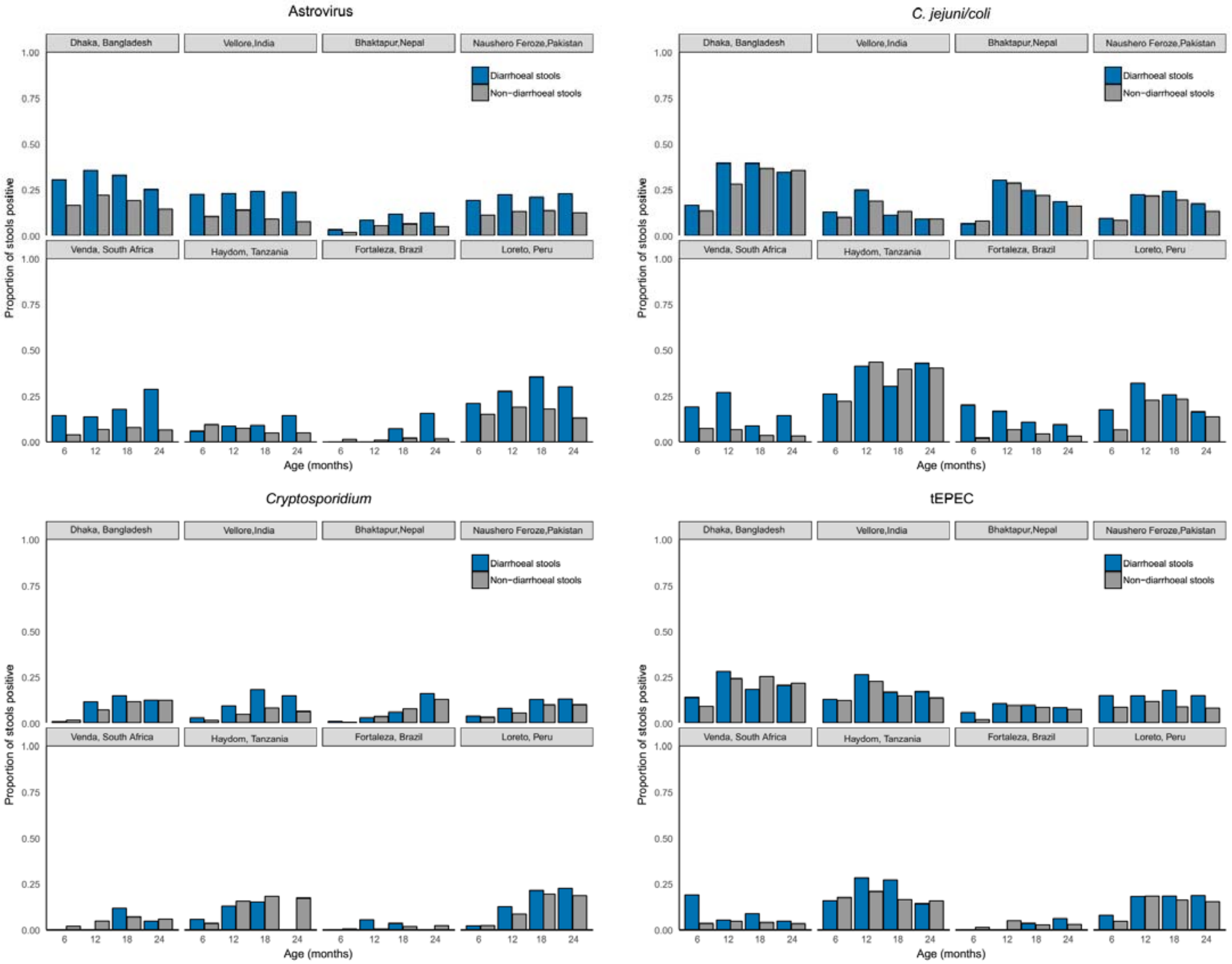
Astrovirus
C. jejuni/coli
Cryptosporidium
tEPEC
Dhaka, Bangladesh
Vellore,India
Bhaktapur,Nepal
Naushero Feroze,Pakistan
Venda, South Africa
Haydom, Tanzania
Fortaleza, Brazil
Loreto, Peru
Diarrhoeal stools
Non−diarrhoeal stools
Proportion of stools positive
Age (months)

Figure S5. Relationship between pathogen quantity and diarrhoea. Pathogens are ordered from left to right and top to bottom according to their prevalence in diarrhoeal cases; the top 20 pathogens by prevalence are shown. The x axis shows pathogen quantity (quantitative PCR Cq; quantity increases from left to right for each pathogen). The lines represent the conditional odds ratios for diarrhoea derived from the regression model as described in the methods except without an interaction between pathogen quantity and age, and the surrounding bands denote the 95% CI. The blue segment of the band, if present, denotes the quantity for which the detection was considered aetiologic (quantity- and episode-specific attributable fraction (AFe) ≥ 0·5). EAEC=enteroaggregative E. coli, EPEC=enteropathogenic E. coli, LT-ETEC=LT-producing enterotoxigenic E. coli, ETEC=enterotoxigenic E. coli, and STEC=Shiga toxin producing E. coli.

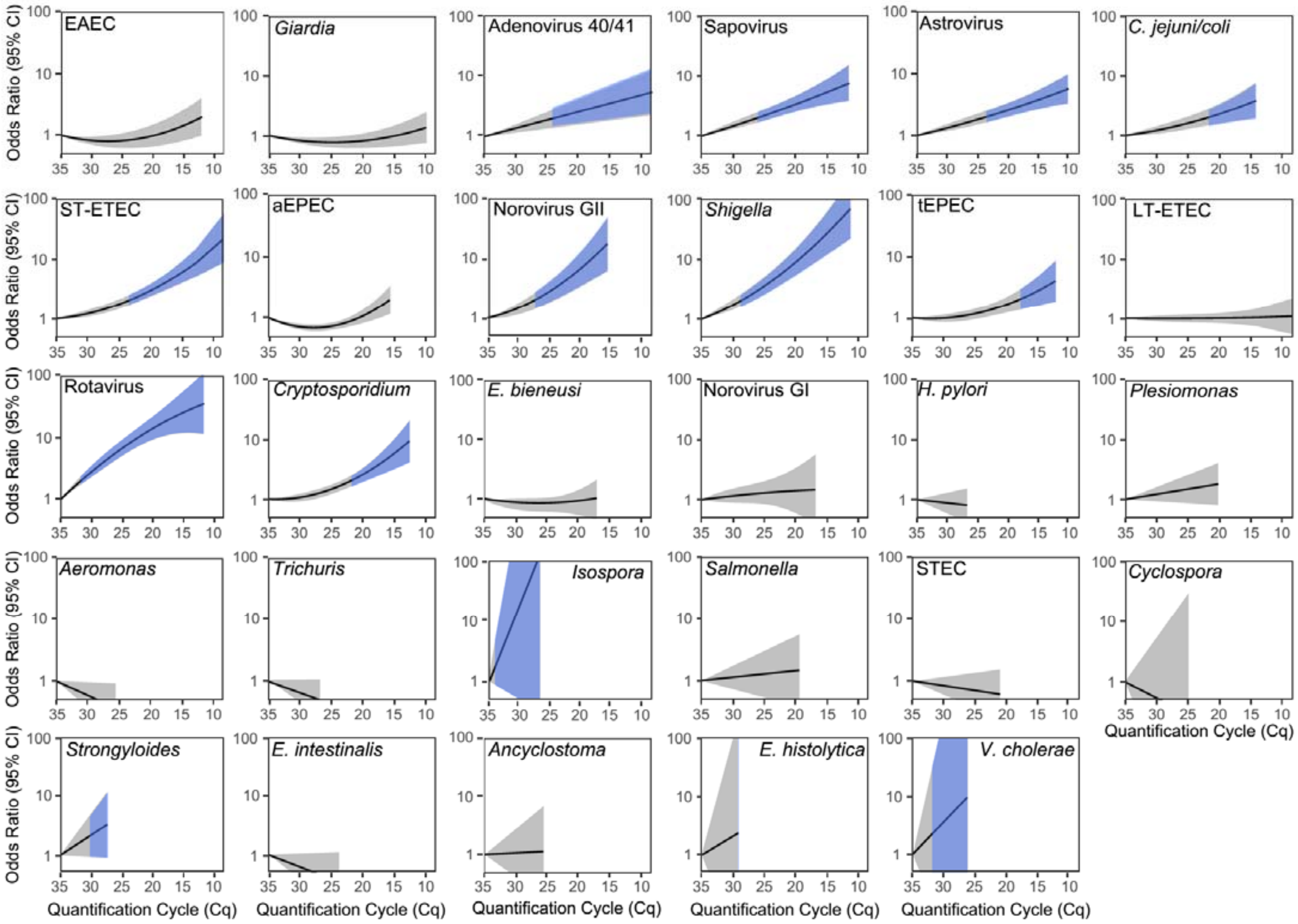

Figure S6. Relationship between pathogen quantity and diarrhoea in children 0-11 months (blue) and 12-24 months of age (red).

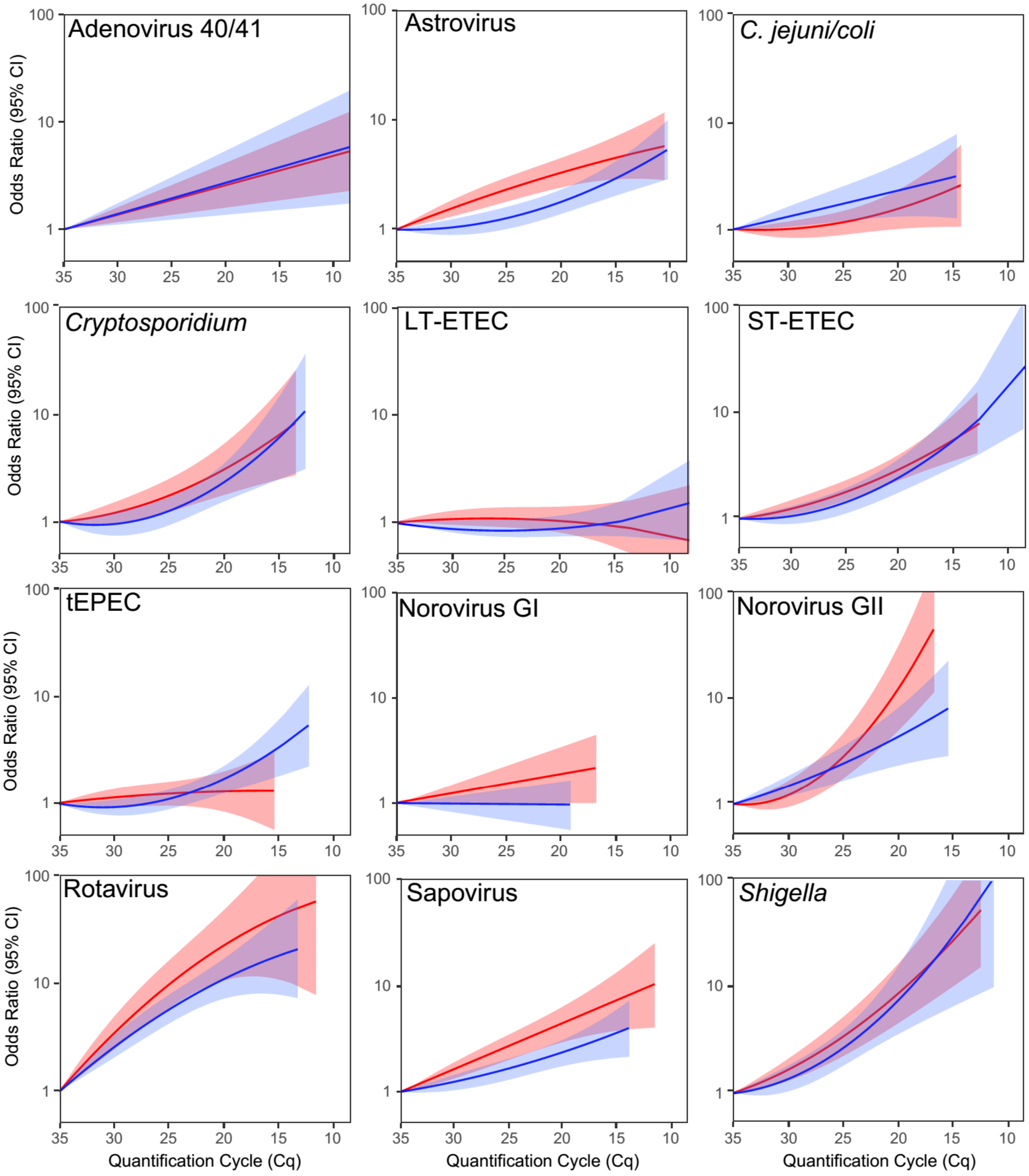

Figure S7. Relationship between pathogen quantity and both severe (red) and non-severe (blue) diarrhoea.

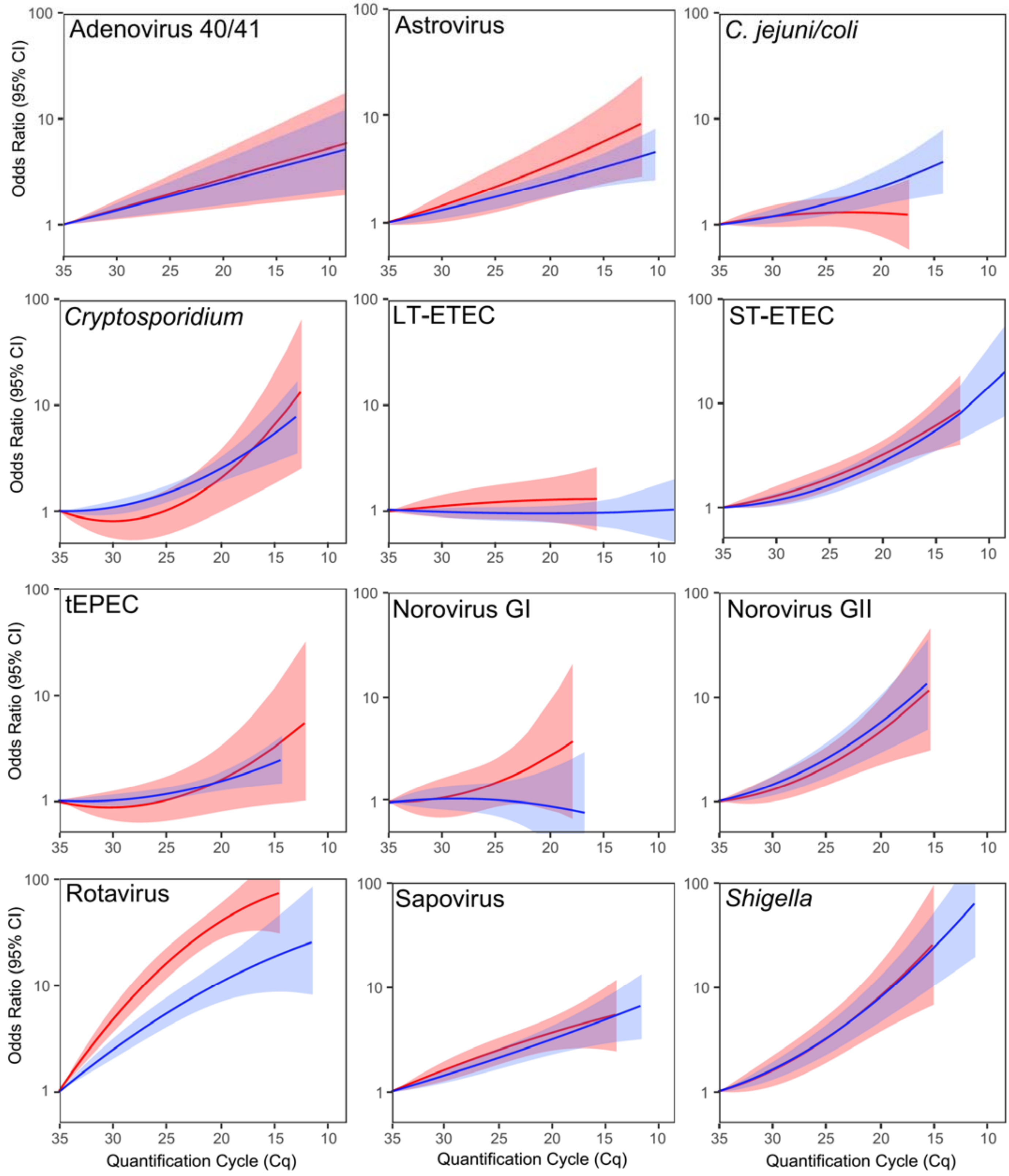

Figure S8. Relationship between pathogen quantity and diarrhoea in MAL-ED (blue) and GEMS (red).

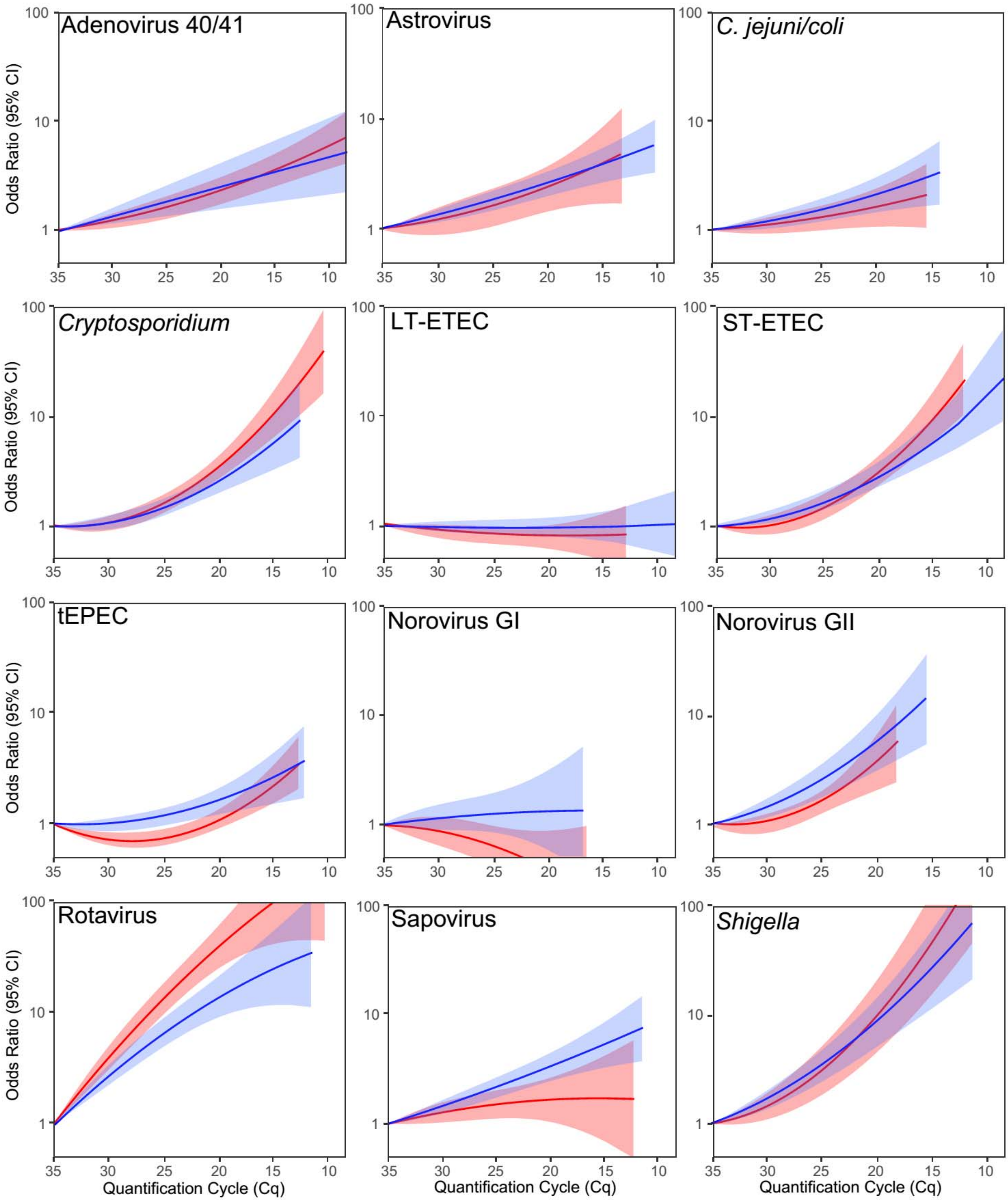

Figure S9. Comparison between estimates of overall attributable incidence by quantitative PCR vs. the original study microbiology. The diagnostic method for the original microbiology is shown in parentheses after each pathogen on the Y axis.

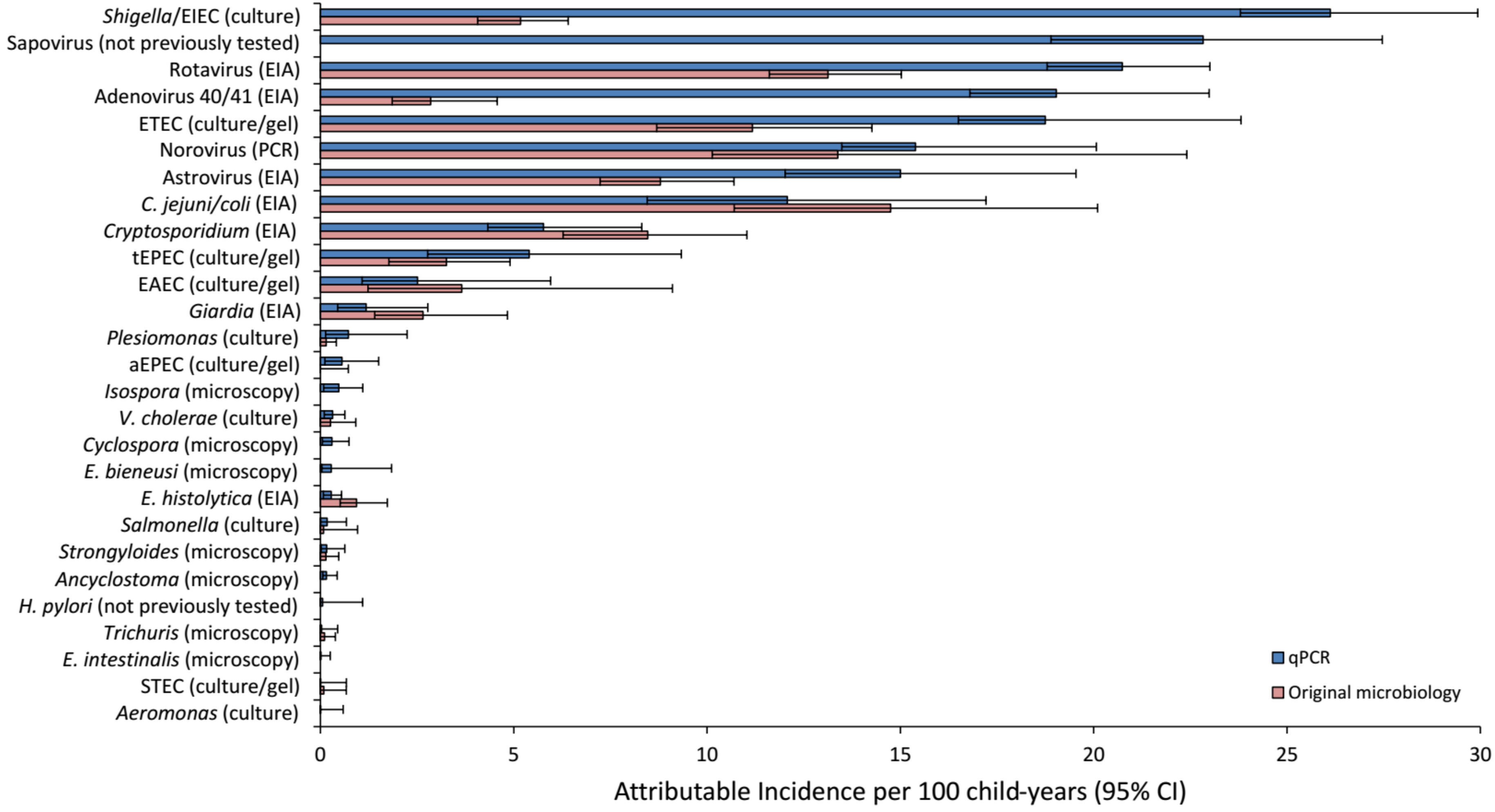

Figure S10. Comparison between estimates of overall attributable incidence by quantification cycle vs copy number. Other than this expression of pathogen quantity, the analyses were identical. Pathogens are ordered by the average attributable incidence between the two metrics.

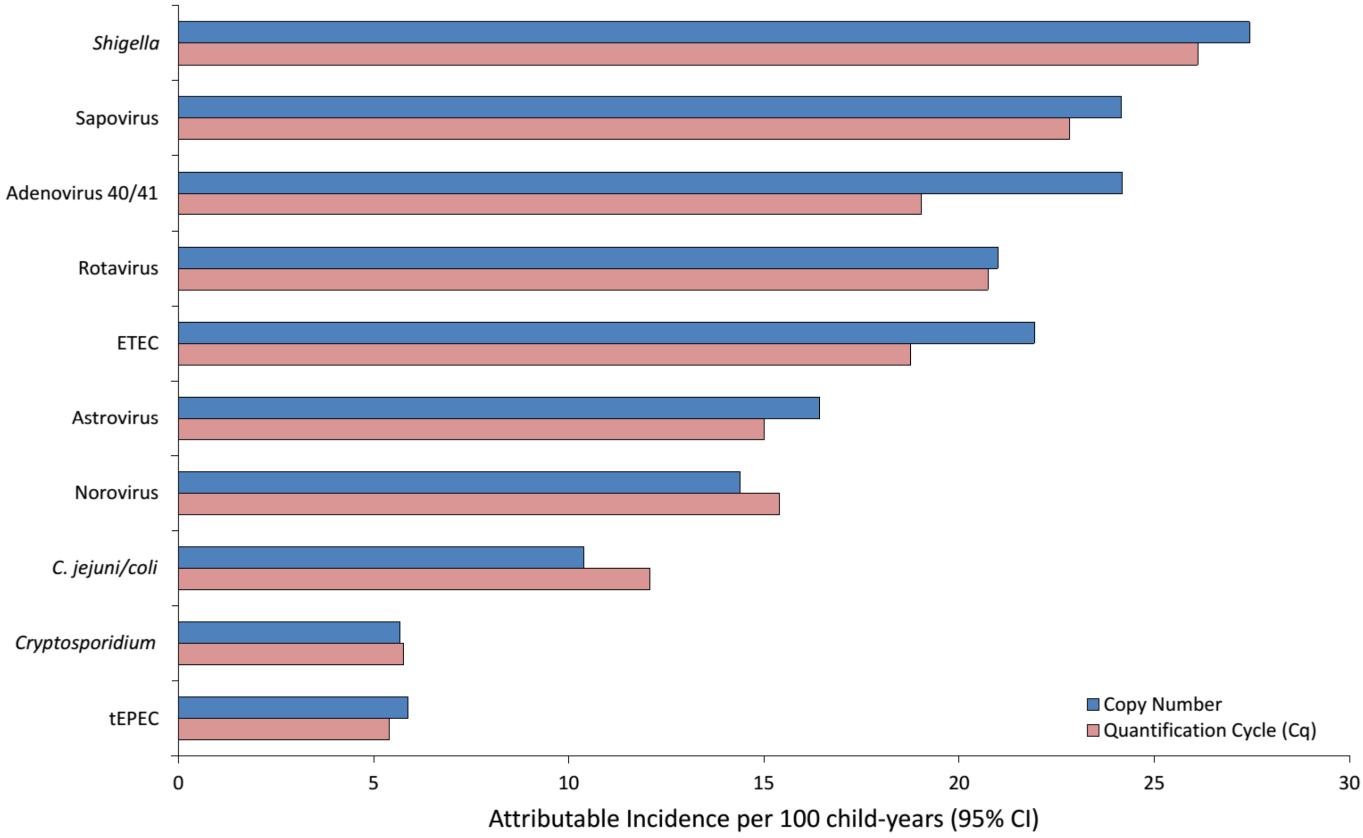

Figure S11. Overall pathogen-specific incidence by attributable fraction method vs. aetiologic detections. Pathogens are ordered by the overall attributable incidence. Aetiologic detections were defined as detections with a pathogen- and quantity-specific AFe (episode attributable fraction) ≥ 0·5.

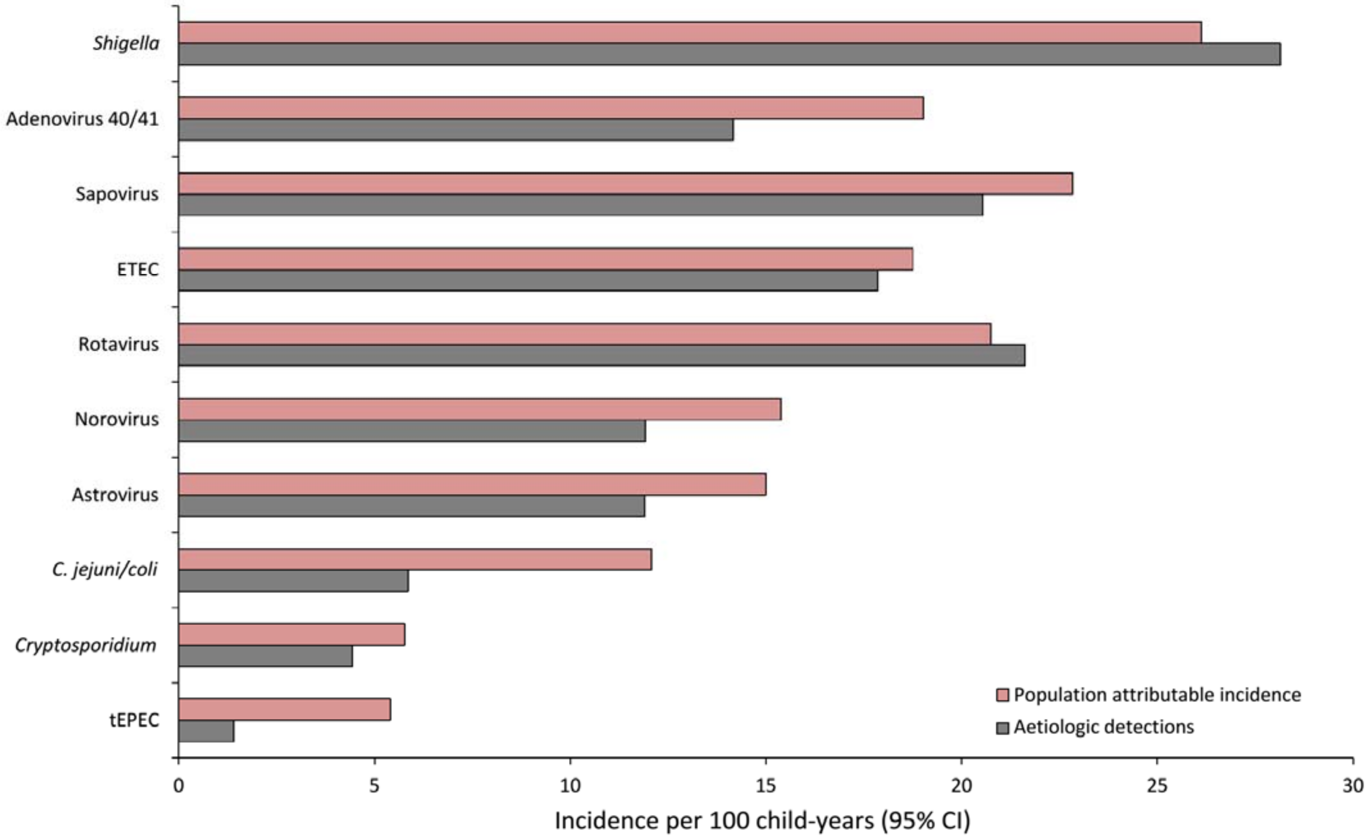

Figure S12. Overall attributable incidence for increasingly restrictive definitions of a non-diarrhoeal stool. This compares the primary analysis, which required non-diarrhoeal stools to be collected at least 7 days before and after surveillance-identified episodes of diarrhoea (blue), with progressively restrictive definitions requiring at least 14 (red) and 28 (grey) days before and after diarrhoeal episodes.

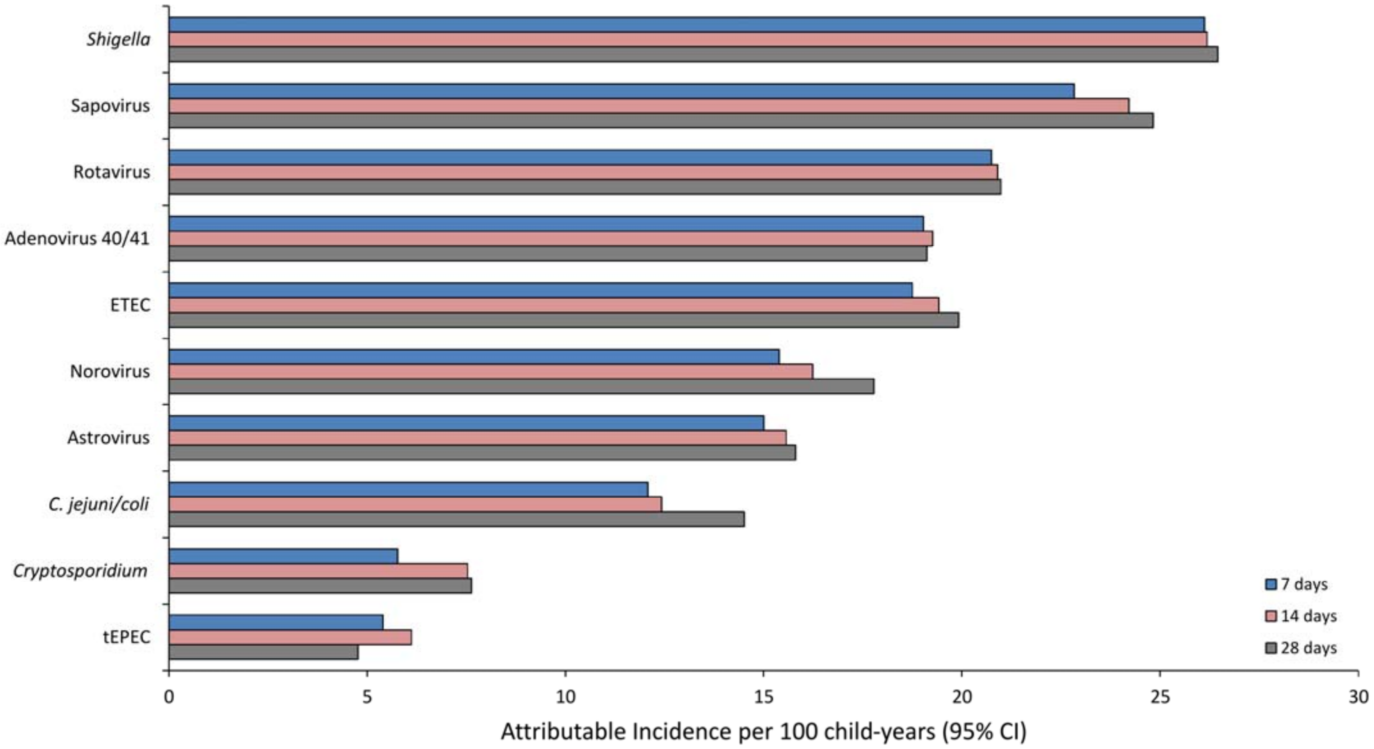

Figure S13. Attributable incidence of diarrhoea with specific clinical characteristics. For each clinical characteristic, the overall attributable incidence was calculated. The ten pathogens with the highest overall burden by qPCR are included and are sorted from left to right by the descending proportion of attributable incidence (plotted on the primary Y axis) in which the characteristic was present (represented by the dotted line, plotted on the secondary Y axis). tEPEC=typical enteropathogenic *E. coli*, ETEC=enterotoxigenic *E. coli*.

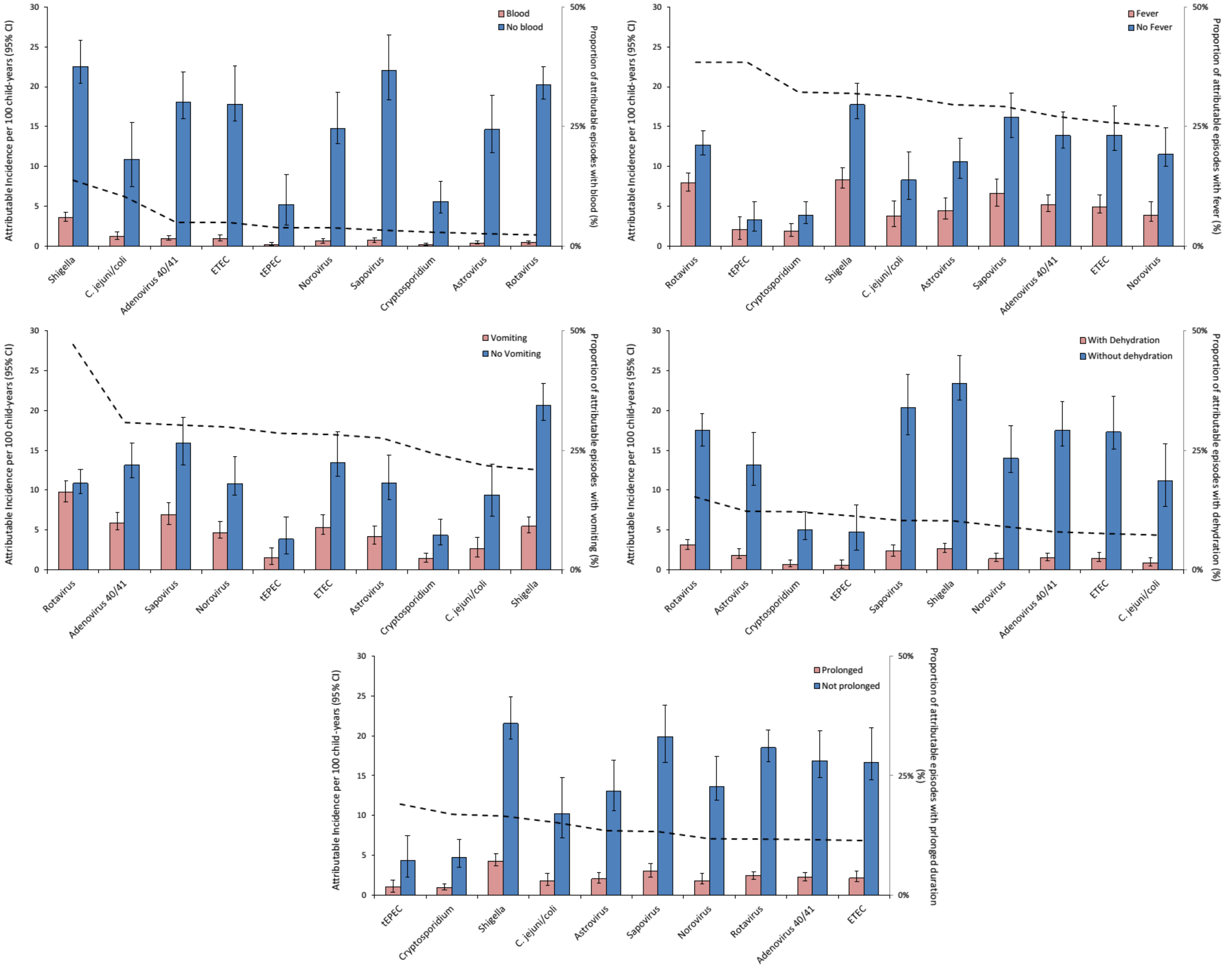

Figure S14. Diarrhoea episodes tested and with primary aetiology identified. Episodes with a primary aetiology were defined as detection of at least one highly diarrhoea-associated pathogen (AFe ≥ 0.5).

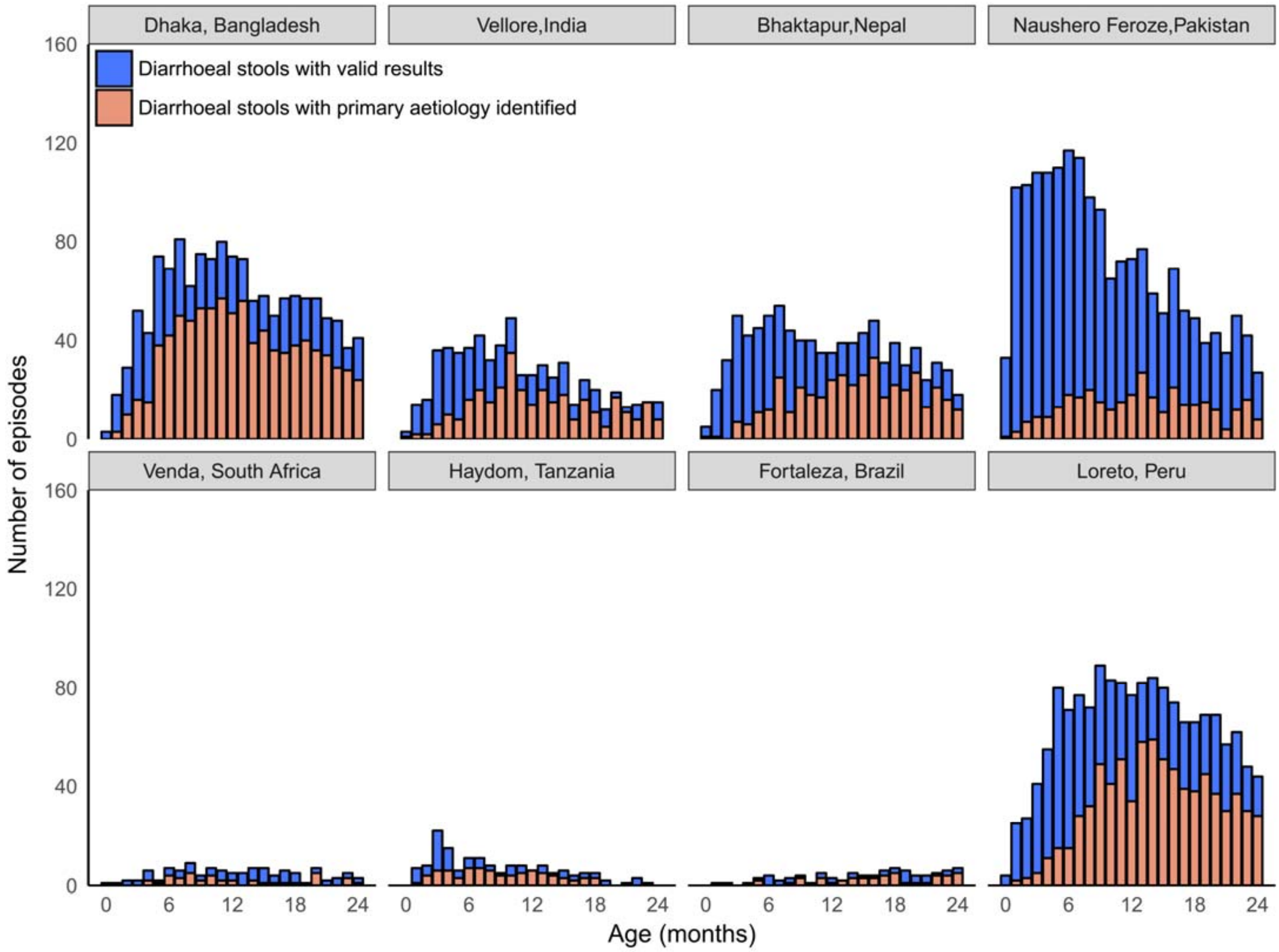

Figure S15. Model-based prediction of aetiology. For each disease, the left plot shows the score distribution by 3-month age intervals. All scores above the blue line, which represents the lowest cut-off which yields greater than 80% specificity, would be considered positive.

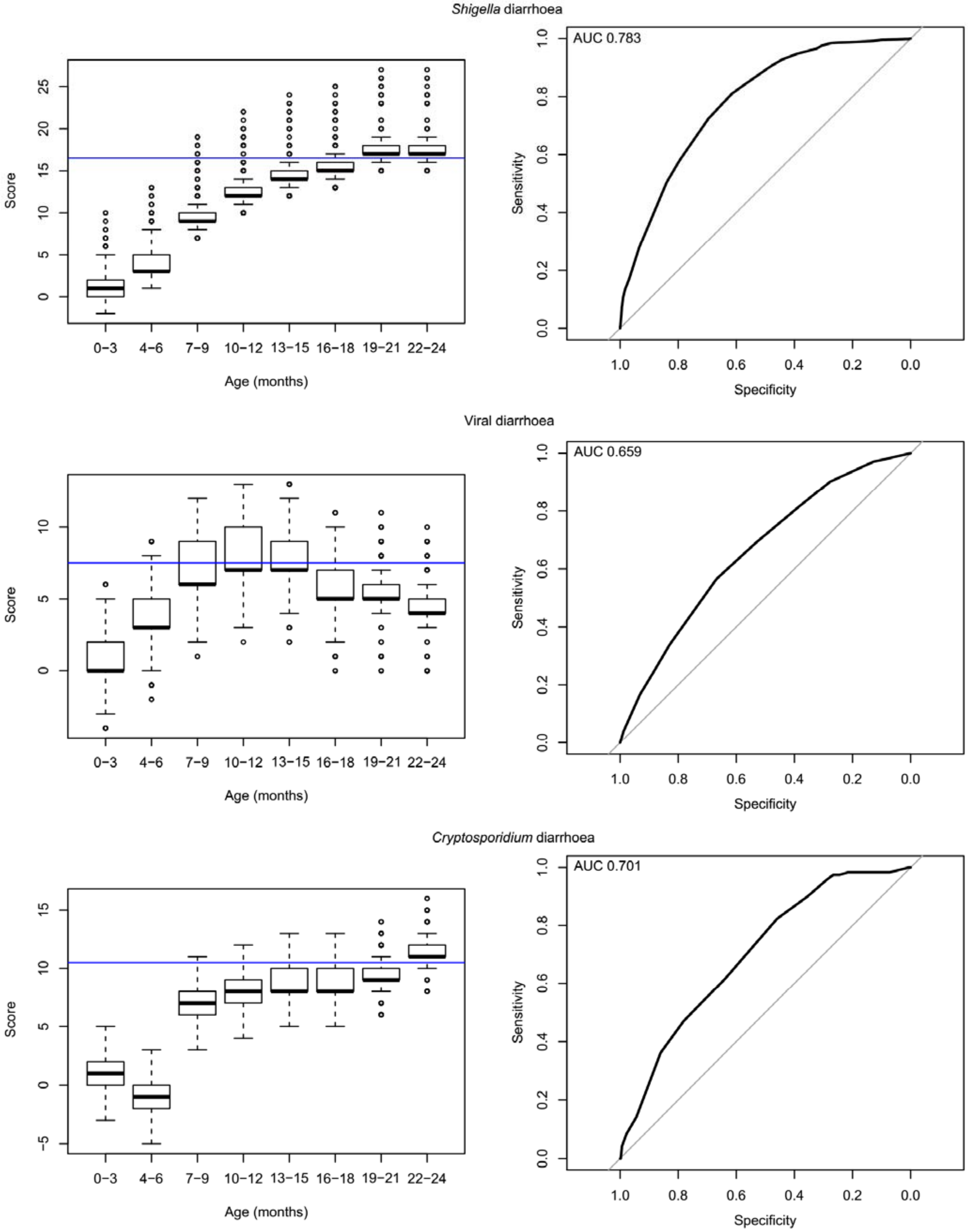

References

1. Liu J, Platts-Mills JA, Juma J, et al. Use of quantitative molecular diagnostic methods to identify causes of diarrhoea in children: a reanalysis of the GEMS case-control study. *Lancet* 2016; **388**(10051): 1291-301.

2. Liu J, Gratz J, Amour C, et al. Optimization of Quantitative PCR Methods for Enteropathogen Detection. *PLoS One* 2016; **11**(6): e0158199.

3. Liu J, Gratz J, Amour C, et al. A laboratory-developed TaqMan Array Card for simultaneous detection of 19 enteropathogens. *J Clin Microbiol* 2013; **51**(2): 472-80.

4. Platts-Mills JA, Liu J, Gratz J, et al. Detection of Campylobacter in stool and determination of significance by culture, enzyme immunoassay, and PCR in developing countries. *J Clin Microbiol* 2014; **52**(4): 1074-80.

5. Platts-Mills JA, Babji S, Bodhidatta L, et al. Pathogen-specific burdens of community diarrhoea in developing countries: a multisite birth cohort study (MAL-ED). Lancet Glob Health 2015; **3**(9): e564-75.

6. WHO. The treatment of diarrhoea: A manual for physicians and other senior health workers. 2005: 44.
